# High sensitivity groups with distinct personality patterns: a person-centered perspective

**DOI:** 10.3389/fpsyg.2024.1336474

**Published:** 2024-08-16

**Authors:** Marcus Bürger, Johann-Christoph Münscher, Philipp Yorck Herzberg

**Affiliations:** ^1^Department of Personality Psychology and Psychological Assessment, Helmut-Schmidt-University, Hamburg, Germany; ^2^German Aerospace Center, Hamburg, Germany

**Keywords:** sensory-processing sensitivity (SPS), environmental sensitivity (ES), latent profile analysis (LPA), sensitivity groups, five-factor model of personality (FFM), personality prototypes, interindividual differences, person-centered analysis

## Abstract

**Introduction:**

Sensory-processing sensitivity (SPS) refers to interindividual differences in sensitivity to positive *and* negative environmental stimuli and reflects the concept of differential susceptibility. The Highly Sensitive Person Scale (HSPS) has been utilized to capture the multifaceted experiences of highly sensitive individuals. The scale’s total score (i.e., the sum of the subfactors) is an indicator of high sensitivity. However, it cannot differentiate between the contributions of the specific subfactors. Consequently, interpreting the total score cannot help resolve the current theoretical debate about how individuals integrate the positive *and* negative aspects of sensitivity, whereas a multidimensional profile should be able to offer a more comprehensive understanding. Intriguingly, in variable-centered research, the subfactors’ differential associations with external constructs in negative *or* positive trait spaces have suggested heterogeneity (i.e., interindividual differences) among highly sensitive individuals. Thus, person-centered approaches should be better suited to address this heterogeneity.

**Methods:**

To explore heterogeneity within the highly sensitive population, we conducted a three-step Latent Profile Analysis in two independent German-speaking samples (*N* = 1,102; *N* = 526). Subsequently, we employed the Five-Factor Model of personality to provide a detailed description of the latent sensitivity groups.

**Results:**

Beyond the frequently identified quantitative three-class differentiation of sensitivity groups, we obtained a four-class model that included two qualitatively different high-sensitivity groups, each displaying distinct HSPS subfactor and personality patterns that corresponded to prototypical personality profiles. Within these high sensitivity groups, (i) the Confident Sensitivity Group exhibited average Neuroticism, significantly above-average Openness, and slightly above-average Extraversion. By contrast, (ii) the Vulnerable Sensitivity Group displayed the typical personality pattern of significantly above-average Neuroticism, below-average Extraversion, and slightly above-average Openness. Personality analyses revealed that features such as passiveness, internalizing tendencies, giftedness, and aesthetics, often commonly ascribed to *all* highly sensitive individuals, are features that differ across distinct sensitivity groups.

**Discussion:**

To avoid over- or underestimating sensitivity effects, future research should consider these interindividual differences in highly sensitive individuals. For instance, studies could focus on the different associations of sensitivity groups with abilities, health aspects, emotion regulation and intervention outcomes, taking into account the different environmental factors that shape the type of sensitivity.

## Introduction

Environmental Sensitivity (ES) refers to the notion that individuals vary in their sensitivity to environmental stimuli, which means that some individuals are more sensitive and some are less so. Scientists propose a genetic basis for sensitivity and suggest that the qualities associated with sensitivity, as well as its impact on the level of functioning, are shaped by valence-dependent contextual experiences (i.e., neutral, harmful/negative, or supportive/positive) throughout life (e.g., Keers and Pluess, 2017). In general, the ES framework proposes five sensitivity types depending on the respective responsivity to positive and negative environments, namely vulnerable sensitivity (i.e., heightened responsivity to negative environments), resilient sensitivity (i.e., resistance to negative environments), differential susceptibility (i.e., heightened responsivity to positive and negative environments), vantage sensitivity (i.e., heightened responsivity to positive environments), and vantage resistance (i.e., resistance to positive environments). These five sensitivity types are covered by different theories, all of which fall under the overarching theoretical framework of ES (see Greven et al., 2019 for a more comprehensive theoretical overview). In particular, early rearing conditions in childhood play a decisive role (Aron et al., 2005; Pluess, 2015; Greven et al., 2019). Thus, early negative environments could lead to a vulnerable-sensitive disposition, while early positive environments likely lead to a vantage-sensitive disposition (Pluess, 2015). The psychological construct of Sensory Processing Sensitivity (SPS) provides a phenotypical and measurable trait of sensitivity with conceptual emphasis on the intense processing of internal and external sensory information (Aron and Aron, 1997). Therefore, the recognition of subtleties, depth of processing, heightened emotional reactivity, and a predisposition to physiological overarousal represent the core definitional elements of the construct (Aron et al., 2012; Homberg et al., 2016). Highly sensitive people (HSP) often view this characteristic as a blessing because it allows them to notice subtle details and aesthetic pleasures in their surroundings. However, it can also be seen as a challenge or a curse, as the effort involved in such processing can drain energy and result in exhaustion (Aron, 2020; Bas et al., 2021; Roth et al., 2023).

Originally, Aron and Aron (1997) developed the Highly Sensitive Person Scale (HSPS) to capture the negative and positive aspects of SPS as a unidimensional construct. Although alternative factor structures have been reported (Konrad and Herzberg, 2017), subsequent exploratory analyses often resulted in a three-factorial structure (Greven et al., 2019), which was first identified by Smolewska et al. (2006) and implemented in the German questionnaire adaptation. The three questionnaire’s subfactors are described as follows (Konrad and Herzberg, 2017):

- The Ease of Excitation (EOE) subfactor operationalizes the tendency to be easily overwhelmed by internal or external stimulation; it captures negative coping strategies such as withdrawal behavior.
- The Low Sensory Threshold (LST) subfactor represents sensitivity or responsiveness to subtle external stimulation, such as sensory features in the physical environment with a low perceptual contrast.
- The Aesthetic Sensitivity (AES) subfactor measures openness to and enjoyment of aesthetic experiences and positive stimuli, reflecting the pleasure gained through high sensitivity.

Numerous studies have provided support for the validity of these subfactors (Liss et al., 2008; Sobocko and Zelenski, 2015; Lionetti et al., 2018; Pluess et al., 2018; Weyn et al., 2021; Pluess et al., 2023). However, they have also suggested that, in the nomological net (Cronbach and Meehl, 1955), the subfactors have distinct associations with external variables. Recently, Attary and Ghazizadeh (2021) explored the nomological net of SPS and identified the obtained trait clusters as positive and negative trait spaces. Accordingly, the negative trait space reflects associations of the EOE and LST subfactors with, for example, Neuroticism (Lionetti et al., 2019; Hellwig and Roth, 2021; Pluess et al., 2023), Negative Affect (Evans and Rothbart, 2009; Yano et al., 2021; Sperati et al., 2024b), alexithymia (Attary and Ghazizadeh, 2021; Jakobson and Rigby, 2021), narcissism (Jauk et al., 2023), negative interpersonal sensitivity (Tabak et al., 2022), and adverse health outcomes such as stress and burnout (Golonka and Gulla, 2021; Pérez-Chacón et al., 2021). Conversely, the positive trait space shows correlations of the AES subfactor with, for example, Openness (Lionetti et al., 2019; Hellwig and Roth, 2021; Pluess et al., 2023), Positive Affect (Evans and Rothbart, 2009; Yano et al., 2021), Effortful Control (Sperati et al., 2024a), lexithymia (Jakobson and Rigby, 2021), positive interpersonal sensitivity (Tabak et al., 2022), proactive work behavior (Schmitt, 2022), and positive health outcomes such as resilience and well-being (Sobocko and Zelenski, 2015; Gulla and Golonka, 2021).

Interestingly, the distinct relationships of the subfactors within the nomological net empirically challenge the notion of a psychological construct that assumed the integration of both the positive *and* negative aspects of high sensitivity in a single individual. In simpler terms, an individual cannot simultaneously exhibit both features (e.g., empathy and alexithymia). Instead, these findings suggest heterogeneity (i.e., interindividual differences), meaning that individuals with higher AES scores are more likely to benefit from their high sensitivity due to their higher level of Openness. Conversely, neurotic individuals with higher scores on LST and EOE may be more vulnerable to stress and overload (Sobocko and Zelenski, 2015). In the same vein, Evans and Rothbart (2008) investigated the latent factor structure of the HSP scale and its relationships with the scales of the Adult Temperament Questionnaire (ATQ, Evans and Rothbart, 2007). They discovered a two-factorial structure that best fitted their data. The first factor primarily predicts EOE items reflecting Negative Affect, highly correlated with Neuroticism (Evans and Rothbart, 2007). Conversely, the second factor is dominated by AES items reflecting Orienting Sensitivity, which correlates with Openness (Evans and Rothbart, 2007). The authors found that Orienting Sensitivity and Negative Affect are orthogonal constructs, each highly correlated with one factor of their two-factorial solution of the HSPS. Moreover, they observed that sensory sensitivity (i.e., perceptual sensitivity in the ATQ) does not correlate with sensory discomfort and, thus, is not inevitably linked to the tendency to experience overarousal. These findings indirectly suggested that the AES and EOE subfactors encompass distinct dimensions.

### Benefits of a person-centered perspective on SPS

When Attary and Ghazizadeh (2021) explored the nomological net of SPS, their analyses included a person-centered perspective, which is an intriguing alternative to previous studies that predominantly used variable-centered methods (e.g., Benham, 2006; Evers et al., 2008; Gearhart and Bodie, 2012; Chacón et al., 2023). Person-centered approaches like Latent Profile Analysis (LPA) are advantageous as they enable the classification of similar objects into groups, where the number of groups and their forms are unknown. In this it is similar to the more traditional and widespread K-means clustering (Magidson and Vermunt, 2002). In contrast to cluster analysis, LPA is model-based, thereby allowing for the consideration of various diagnostics to determine the number of clusters (i.e., model fit indices like the Bayes Information Criterion, BIC). Furthermore, it allows the description of misclassification and the inclusion of external variables (Magidson and Vermunt, 2002). In variable-centered SPS research, researchers assess the highly sensitive population characteristics and their interactions based on linear covariation of a set of individual variables. This analytic approach assumes that the results apply to the entire population. Moreover, it isolates each individual observation in the sample (Mandara, 2003) without adequately accounting for the multidimensional and interactional nature of the phenomenon (Magnusson, 2001). Instead, person-oriented approaches allow interindividual variance to be considered, thereby increasing the predictive power and specificity of the derived description (Mandara, 2003). Similarly, the resulting trait patterns provide a more comprehensive and ecologically valid understanding of interindividual human experiences (Mandara, 2003). Therefore, person-centered approaches are better suited to investigate the integration of both the negative *and* positive trait aspects by exploring latent groups with common SPS trait patterns (De Gucht et al., 2022). Additionally, the derived latent groups can be further described by incorporating external personality characteristics (Mandara, 2003; Berlin et al., 2014) provided by, for example, the Five-Factor Model of personality (FFM).

The few person-centered studies that have been conducted in the area of SPS have contributed to the understanding of the construct in three key ways. First, these studies have consistently demonstrated the quantitative differentiation of sensitivity groups, categorizing HSP into three categories: dandelions (low sensitivity group), tulips (intermediate sensitivity group), and orchids (high sensitivity group) in adults (Lionetti et al., 2018; May et al., 2020; Yano and Oishi, 2021), adolescents (Tillmann et al., 2021), and children (Pluess et al., 2018). Second, researchers have used external variables in combination with the HSPS to examine latent sensitivity groups. These analyses involve personality traits beyond SPS to explore how SPS interacts with other external personality constructs such as alexithymia (Jakobson and Rigby, 2021) and narcissism (Jauk et al., 2023). In order to provide valuable insights into potential behavioral dynamics (e.g., with regard to withdrawal tendencies in response to excessive demands), such studies have focused on the negative trait space of SPS. Third, Aron and Aron (1997) reported two qualitatively distinct clusters observed in three independent samples. One highly sensitive group exhibited a well-adjusted trait profile indicating resilience, whereas the other group displayed greater maladjustment indicating vulnerability, characterized by high emotionality and introversion. The authors suggested that the latter personality pattern might arise due to an early-forming insecure attachment style stemming from a troubled childhood, combined with a genetic predisposition to sensitivity. In contrast to the vulnerable sensitivity group, the well-adjusted group demonstrated the opposite pattern, displaying more emotional stability and reporting happier childhood experiences. In essence, these different sensitivity groups reflected patterns in the personality domains of Neuroticism and Extraversion (Eysenck, 1991).

Broadening the person-centered personality perspective to include all five FFM domains, beyond just Neuroticism and Extraversion (e.g., Aron and Aron, 1997), allows for the consideration of research on prototypical personality patterns (Rammstedt et al., 2004; Block and Block, 2006; Herzberg and Hoyer, 2009; Kerber et al., 2021). To date, three prototypical personality profiles have consistently been replicated: the Undercontroller type, the Overcontroller type, and the Resilient type (Kerber et al., 2021). These prototypes are based on the theory of ego-control and ego-resiliency (Block and Block, 2006). Additionally, three other types have been identified in research, though less frequently: Reserved, Confident, and Non-desirable (Kerber et al., 2021). These personality prototypes exhibit characteristic FFM domain profiles that show predictive power for various psychological variables such as locus of control, self-esteem, well-being, and health (Kerber et al., 2021). If sensitivity groups with different domain profiles correspond to prototypical personality patterns in the current study, it will provide an opportunity to compare the findings with empirical evidence from this research area, offering a novel differential perspective in SPS research.

### Associations between SPS and the five-factor model of personality

The FFM provides a comprehensive and well-supported framework for describing personality constructs in the nomological net (Bainbridge et al., 2022). Studies investigating relationships between personality and SPS have consistently identified that the HSPS total score is strongly correlated with Neuroticism and Openness. Upon considering the HSPS subfactors, both the EOE subfactor and the LST subfactor were significantly positively correlated with Neuroticism (negative trait space), whereas the AES subfactor demonstrated a significant positive correlation with Openness (positive trait space) (Greven et al., 2019; Lionetti et al., 2019). Among the remaining three FFM domains (i.e., Extraversion, Agreeableness, and Conscientiousness) correlations with SPS were less consistent, but they could still contribute to describing interindividual differences among highly sensitive individuals (for a comprehensive review of previous association studies, see Greven et al., 2019). Extraversion appears particularly relevant for describing interindividual differences, given its often differential relationships with the subfactors. The LST and the EOE subfactor tend to show significant negative correlations with Extraversion, whereas the AES subfactor exhibits significant positive correlations (Hellwig and Roth, 2021; Pluess et al., 2023). However, in a meta-analysis, the associations appeared to be lower and non-significant (*r*EOE = −0.05, ns; *r*LST = −0.07, ns; *r*AES = 0.08, ns), which may be attributable to the combined analyses with the Behavioral Activation System (Lionetti et al., 2019). Consequently, some highly sensitive individuals may express their sensitivity in an extraverted manner, whereas others exhibit more introverted behaviors (Aron and Aron, 1997; Aron, 2020).

The FFM domains can be further broken down into six facets each, providing a more nuanced overview of personality (Goldberg, 1999). Studies examining the relationship between personality facets and SPS are scarce but valuable, as they can uncover null associations at the domain level that may be explained by contradictory correlations at the facet level (Greven et al., 2019). For instance, Pluess et al. (2023), who used the International Personality Item Pool (Goldberg, 1999), showed that the EOE subfactor has no significant correlation with the Openness domain (*r* = −0.06, ns). However, Openness facets such as Imagination (*r* = 0.16, *p* < 0.01) and Adventurousness (*r* = −0.43, *p* < 0.01) present opposing associations that contribute to the overall domain score. Only three studies to date have included all 30 facets in their examinations (Bröhl et al., 2020, 2021; Pluess et al., 2023). Of these, one study included self-identified highly sensitive adults (Bröhl et al., 2021). This study found that six facets were linked to SPS: Anxiety (N1), Depression (N3), Aesthetics (O2), Fantasy (O1), Feelings (O3), and Gregariousness (E2). On the basis of these findings, the authors concluded that highly sensitive individuals lean toward internalizing tendencies, are sensitive to aesthetics, and exhibit passivity. Additionally, the authors argued that interindividual differences exist among highly sensitive people concerning general (mal-)adjustment due to the only moderate interrater agreement between the ratings of the FFM facets of Agreeableness and Conscientiousness domains. Therefore, these facets did not show significant associations with the HSPS total score but varied among highly sensitive participants.

Therefore, we investigated whether different types of highly sensitive individuals can account for the heterogeneity observed in empirical studies. For this purpose, we utilized data-driven, person-centered analyses at the subfactor level (AES, LST, and EOE) to explore latent sensitivity groups, although the HSPS was not originally constructed with such an intention (Aron et al., 2012). Subsequently, we characterized these sensitivity groups on the basis of their emerging personality patterns. We anticipated the emergence of qualitatively distinct subfactor patterns that reflect the heterogeneity (i.e., interindividual differences) of highly sensitive individuals, as dictated by the unique associations of the negative trait space subfactors (EOE/LST) and the positive trait space subfactor (AES). Moreover, we expected that the sensitivity groups might differentiate individuals located within the low and medium sensitivity trait ranges. However, in this study, we focused on the higher sensitivity trait range, assuming that behavior and trait conceptualizations are aligned in this part of the distribution, in the sense of traitedness (Reise and Waller, 1993).

Thus, we proposed five sensitivity groups:

- a group characterized by medium to low scores on the AES subfactor and high scores on the EOE/LST subfactors (maladapted sensitivity group);
- conversely, a group with medium to low scores on the EOE/LST subfactors and high scores on the AES subfactor (well-adjusted sensitivity group);
- a third group featuring high scores on both trait space (EOE/LST and AES), aligning with the notion of differential susceptibility (‘for better *and* for worse’);
- a fourth group with low scores on all three subfactors (low sensitivity group);
- lastly, a group with medium scores, representing the average levels of model indicators across the sample (medium sensitivity group).

## Methods

### Participants and procedure

For the purpose of cross-validation (Koul et al., 2018), we used two samples (A and B) from independent personality research projects targeting the construct validation of SPS (titled: “High sensitivity and personality”). Both studies were conducted approximately from 2016 to 2019. We placed study announcements on websites that offer information on SPS in German-speaking countries and recruited most participants there. Furthermore, we invited students from our university to participate. Both web-based samples contributed to the only available German norm sample (Herzberg et al., 2022). Utilizing a unified rule that was applied to create participants codes to anonymize the data, we ensured that participants who participated in both studies were eliminated from Sample B while remaining in Sample A (applied to *N* = 368 participants). After the data were prepared, Sample A comprised *N* = 1,102 and Sample B *N* = 536. In Sample A participants’ age ranged from 18 to 71 years (*M* = 39.3, *SD* = 11.14), whereas in Sample B age ranged from 16 to 70 (*M* = 40.2, *SD* = 11.55). In Sample B, five participants were between the ages of 16 and 17. We decided to keep their HSPS-G responses in the overall adult sample as the results were unaffected in the multigroup analyses of the NEO-PI-R (McCrae et al., 2005a,b), and the proportion of the sample seemed neglectable. Table 1 presents the frequencies of sex, education and employment groups. A prerequisite for participation was the ability to answer web-based self-reports using technical devices (e.g., smartphones or PC). In line with the declaration of Helsinki, participants gave informed consent at the beginning of the survey. Students from our university received course credit if required.

**Table 1:** Demographics of Sample A, Sample B, and total sample.

		Sample A		Sample B		Total	
Variable	Response	*n*	%	*n*	%	*n*	%
Sex	Men	171	15.5	59	11.2	230	14.1
	Women	931	84.5	467	88.8	1,398	85.9
Education	Primary education	96	8.7	60	11.4	156	9.6
	Secondary education	435	39.5	221	42.0	656	40.3
	Higher (academic) education	571	51.8	245	46.6	816	50.1
Employment	Yes, self-employed	181	16.4	77	14.6	258	15.8
	Yes, employee	554	50.3	258	49.0	812	49.9
	No, school or university student	151	13.7	86	16.4	237	14.6
	No, retired	43	3.9	28	5.3	71	4.4
	No, homemaker	68	6.2	32	6.1	100	6.1
	No, unemployed jobseeker	105	9.5	45	8.6	150	9.2

Primary education refers to schooling up to 10 years; Secondary education encompasses consecutive schooling or vocational education (often additional 3 years); Higher education denotes academic attainment (i.e., achieving a Bachelor’s degree, a Master’s degree, a PhD, or the status of master craftsman).

### Measures

#### The NEO-PI-R

We used the German version of the NEO-PI-R personality inventory (Ostendorf and Angleitner, 2004) to assess the five FFM personality domains (i.e., Neuroticism, Extraversion, Openness, Agreeableness, Conscientiousness) and 30 FFM personality facets (i.e., six facets nested in each domain). Table 2 presents an overview of the FFM facet description alongside internal consistency measures (i.e., Cronbach’s alpha). The German version was adapted from the original English version (Costa and McCrae, 1992).

**Table 2:** Overview of the NEO-PI-R facets (Description for high scorers on the respective facet).

	Domains and facets	Description	Cronbach’s alpha	
			Manual	Current
				
	(N)euroticism		0.92	0.93
N1	Anxiety	Worry and physiological reactions to anxiety	0.81	0.81
N2	Angry Hostility	Readiness to becoming angry and annoyed, and being temperamental	0.77	0.73
N3	Depression	Self-blame, loneliness, self-confidence/−worth, sadness, and hopelessness	0.82	0.85
N4	Self-Consciousness	Embarrassment and lack of self-worth	0.69	0.77
N5	Impulsiveness	Giving in to cravings and difficulties of restraining and controlling oneself	0.65	0.67
N6	Vulnerability	Reduced coping efficacy and weaker emotional stability	0.80	0.81
	(E)xtraversion		0.89	0.89
E1	Warmth	Cordially, approachable, strong bonds with friends	0.74	0.75
E2	Gregariousness	Enjoying crowds and big social gatherings	0.77	0.81
E3	Assertiveness	Dominance, assertiveness and leadership behavior	0.80	0.80
E4	Activity	Lively, fast-paced work and life, vigorous	0.74	0.70
E5	Excitement-Seeking	Seeking crowds at big events and scary movies, action and adrenaline chasing	0.66	0.66
E6	Positive Emotions	Positive affect: joyful, cheerful, light-hearted, optimistic	0.79	0.82
	(O)penness to Experience		0.89	0.87
O1	Openness to Fantasy	Active imagination and an affinity for daydreaming	0.79	0.75
O2	Openness to Aesthetics	Enjoying, fascination of, and interest in music and art	0.79	0.75
O3	Openness to Feelings	Experiencing strong emotions, appreciation and recognition of emotions	0.75	0.73
O4	Openness to Actions	Trying new methods and ways, and willingness to experience new surroundings	0.66	0.72
O5	Openness to Ideas	Affinity to philosophical and abstract theories, ideas, and discussions, as well as an intellectual interest	0.81	0.77
O6	Openness to Values	Tolerance for other societies’ idea of right and wrong, and open-mindedness to different believes	0.49	0.49
	(A)greeableness		0.90	0.86
A1	Trust	Trustful, believes in the best of people	0.79	0.81
A2	Straightforwardness	Reluctance to manipulate people and aversion to be called a hypocrite	0.69	0.54
A3	Altruism	Concerns for others, e.g., being considerate and generous	0.74	0.67
A4	Compliance	Cooperation, restraint in negative emotion expression, flexible	0.70	0.62
A5	Modesty	Bottom-up comparison to others, lower opinion of oneself, and the reluctance to talk about oneself	0.75	0.74
A6	Tender-Mindedness	Social, sympathy for others	0.68	0.64
	(C)onscientiousness		0.93	0.88
C1	Competence	Self-efficacy and feeling of control over one’s life	0.71	0.62
C2	Order	Tidy, organized, neat, demanding	0.73	0.68
C3	Dutifulness	Conscientious in performing tasks, dependable, reliable, adhering to principles	0.75	0.62
C4	Achievement Striving	Working towards goals, drive to get ahead and excel	0.71	0.59
C5	Self-Discipline	Productive, persevering, even when dealing with a big workload	0.84	0.82
C6	Deliberation	Consideration during decision making and planning process	0.77	0.78

#### The highly sensitive person scale

The 26-item German version of the Highly Sensitive Person Scale (HSPS-G) (Konrad and Herzberg, 2017) contains three subfactors: Ease of Excitation (EOE, 10 items, Cronbach’s *α* = 0.82), Low Sensory Threshold (LST, 11 items, Cronbach’s *α* = 0.88) and Aesthetic Sensitivity (AES, five items, Cronbach’s *α* = 0.65). Cronbach’s *α* for the HSPS-G total score = 0.91 (information on reliability refers to the current study). Participants rated statements on a 5-point Likert scale (ranging from (0) *does not apply to me at all* to (4) *applies to me completely*).

### Data analysis

#### Data preparation

Following the preanalytical suggestions in the NEO-PI-R manual, we first considered responses to the two single, self-reported attention and effort items presented at the end of the NEO-PI-R questionnaire (“*I have made every effort to answer all questions honestly and accurately*” and “*Did you tick all the answers in the right places?*”). Second, we considered several preanalytical screening methods (Yentes and Wilhelm, 2021) because web-based data from totally anonymous participants seem particularly susceptible to careless responding (Meade and Craig, 2012). We employed the “longstring” function to rule out identical response behavior regarding consecutive items. Furthermore, we used Mahalanobis distance to detect multivariate extreme values (*p* < 0.001). For this purpose, we considered all three HSPS-G subfactors and the five NEO-PI-R domain scores. Finally, the acquiescence value indicated how many participants conspicuously responded in terms of frequent confirmations and refusals (Ostendorf and Angleitner, 2004). First, we registered 19 invalidating responses on the NEO-PI-R attention and effort items. Second, 21 participants showed suspicious acquiescence behavior. Third, we applied the Mahalanobis Distance criterion to 12 subjects and detected “longstring” behavior four times. Finally, we excluded one participant due to invalid age information. The participants presented overlap regarding the exclusion criteria. Therefore, we excluded 49 subjects out of 1,151 from Sample A. In order to comply with open science recommendations, our data and the LG syntax are available at https://osf.io/tycr9/?view_only=2a9a02c59f3c433592d95f4319dd82e8.

#### Latent profile analysis

Latent profile analysis (LPA) is a probabilistic, model-based, exploratory procedure that estimates class-dependent conditional response probabilities (CRPs) and class proportions. The expectation–maximization algorithm estimates model parameters while maximizing the likelihood estimation (Masyn, 2013). In our study, we used a bias-adjusted three-step approach (Vermunt, 2010; Masyn, 2017). The three steps involved (i) estimating the model, (ii) assigning participants to the latent groups (i.e., sensitivity groups) on the basis of the individual posterior class probabilities, and (iii) investigating the associations between the assigned class memberships and the external variables (i.e., NEO-PI-R domains and facets in our study). We used Latent Gold (version 6) as statistical software.

The first step was model estimation, for which we used the three HSPS-G subfactors (AES, EOE, and LST) as model indicators because of the empirical evidence outlined above. We applied this first step of the LPA to two independent samples (Samples A and B) to cross-validate the latent group structure. Subsequently, we merged the two samples into a total sample, and we also applied the first step of the LPA to generate the final latent model. Thus, we increased the confidence in the estimated parameters (i.e., reliability) by considering all available observations (i.e., participant responses). Below, we report the model estimation and selection procedures separately in the results section (i.e., the first step of the LPA), including the interpretation of all model fit indices.

As a result of the first step of the LPA, individuals obtain posterior class probabilities for each sensitivity group ranging from 0 to 1 (i.e., proportional group assignments). The group memberships are frequently imperfect or ambiguous (i.e., they deviate from 1), indicating a potential classification error (CE) when modal class assignments are employed. The CEs have a complementary relationship with the sensitivity groups’ Average Posterior Probability (AvePP), such that AvePP + CE equals 1 (Bauer, 2022). Once the proportional group assignment (i.e., individual class probabilities) gets translated into a modal class assignment by using the highest class probability, a bias in the associations with external variables is imposed (i.e., FFM of personality in this study). For example, say we assume a 4-class model in which an individual’s set of class probabilities of [0, 0, 0.2, 0.8] translates into a modal class assignment of [0, 0, 0, 1]. In this situation, the modal assignment would neglect the imprecision of the class assignments for Groups 3 and 4 and simply allocate the individual to the fourth sensitivity group, thus creating biases. Effect sizes are generally underestimated when a modal class assignment is employed (Vermunt, 2010).

Hence, as a third LPA step, we investigated the unbiased associations of the sensitivity groups with the NEO-PI-R domains and facets. For the end of bias adjustment implementation, we applied the BCH procedure (Bolck et al., 2004) to correct the CE in participants of Sample A, for which we had that data on NEO-PI-R responses. To interpret meaningful results, we relied on the statistical significance of the model parameters and deviations from the sample mean (displayed by the Medium Sensitivity Group) that at least indicate small effect sizes (i.e., *z*-values ≥0.2) in terms of Cohen’s *d* (Cohen, 1992).

#### Model estimation and selection

When estimating the models, we customized the number of iterations and random seeds because the default mode seemed insufficient, as it is suspected of identifying local maxima as the best model solution (Berlin et al., 2014). Accordingly, we set 10,000 random seeds and 500 iterations with the convergence criteria remaining as predefined (1e^−08^). Berlin et al. (2014) recommend running the estimation process as many times as necessary to achieve an equal final-stage solution at least two times. We followed these recommendations to raise confidence about the stability of our models.

An essential part of model selection in LPA involves determining the number of latent classes. As many scholars have proposed, we based our decision on a joint and well-balanced consideration of several well-supported model fit indices (i.e., local and relative fit indices), information criteria (IC), and substantive or meaningful interpretability of obtained classes (Nylund et al., 2007; Schreiber, 2017; Nylund-Gibson and Choi, 2018). Bivariate Residuals (BVRs) reflect local fit, yielding information on the models’ conformity with the local independence assumption (Asparouhov and Muthén, 2015; Oberski, 2016). Bayesian Information Criterion (BIC), Akaike’s Information Criterion (AIC), and Approximate Weight of Evidence (AWE), amongst others, provided information about the models’ parsimony. The Integrated Completed Likelihood (ICL-BIC) contains information on BIC, additionally considers Entropy (Biernacki et al., 2000), and provides a trade-off between the two fit indices. The Vuong-Lo–Mendell–Rubin Likelihood Ratio Test (VLMRT) and Parametric Bootstrap Likelihood Ratio Test (BLRT) assess relative model fit. Eventually, we considered the CE (i.e., 1 - accuracy) and smallest class size. The probability of finding reliable classes potentially valid in the population drops with smaller class sizes (Nylund-Gibson and Choi, 2018). Some experts have proposed a 5 % threshold, but general recommendations on this topic do not exist. Another indicator describing the consistency of individual class assignments is the average posterior probability (AvePP). Values above 0.70 indicate well-separated classes (Nagin, 2005). The Entropy provides a condensed measure of classification accuracy and should be at least 0.6, while values between 0.8 and 1.0 are considered desirable (Bauer, 2022).

## Results

### Full sample description

The correlational results at the domain level presented in Table 3 were in line with previous empirical findings (e.g., Lionetti et al., 2019; Pluess et al., 2023). As anticipated, not only were the associations of AES and EOE with the FFM domains the highest but they were also distinct, indicating different interindividual trait patterns. When examining the relationships between the HSPS scales and FFM domains, the highest correlations were observed between AES and Openness (*r* = 0.52, *p* < 0.01) and EOE and Neuroticism (*r* = 0.51, *p* < 0.01). These differential relationships were also evident when comparing the correlations with Neuroticism (*r*_AES_ = 0.10, *p* < 0.01; *r*_EOE_ = 0.51, *p* < 0.01) and Openness (*r*_AES_ = 0.52, *p* < 0.01; *r*_EOE_ = 0.07, *p* < 0.05). A similar pattern emerged for Extraversion, with correlations with AES (*r* = 0.05, ns) and EOE (*r* = −0.43, *p* < 0.01). Agreeableness, on the other hand, consistently showed significant links with both HSPS-G subfactors (*r* = 0.17, *p* < 0.01). It’s worth noting that the contrast between AES (in the positive trait space) and the other two subfactors, EOE/LST (in the negative trait space), was smaller for LST than for EOE.

**Table 3:** Descriptives and bivariate correlations of sex, age, HSPS subfactors, and NEO-PI-R domains (Sample A).

	M	SD	1	2	3	4	5	6	7	8	9	10
1. Sex												
2. Age	39.30	11.14	−0.01									
3. AES	16.48	2.82	0.19**	0.12**								
4. EOE	29.61	6.43	0.21**	0.11**	0.37**							
5. LST	29.60	8.16	0.32**	0.20**	0.50**	0.63**						
6. SPS	75.68	14.77	0.30**	0.18**	0.63**	0.85**	0.92**					
7. N	109.29	25.54	0.17**	−0.12**	0.10**	0.51**	0.30**	0.41**				
8. E	89.83	21.48	0.03	−0.13	0.05	−0.43**	−0.23**	−0.30**	−0.31**			
9. O	131.23	18.07	0.17**	−0.02	0.52**	0.07*	0.23**	0.26**	0.01	0.31**		
10. A	122.01	16.96	0.18**	0.10**	0.17**	0.17**	0.16**	0.19**	−0.06	0.02	0.28**	
11. C	118.76	19.70	−0.02	0.04	0.06*	−0.06	0.01	−0.01	−0.30**	0.02	−0.03	0.09**

*N* = 1,102. *p < 0.05; **p < 0.01. Only correlations of ‘Sex’ are non-parametric (Spearman); Male sex is coded = 1 and female sex is coded = 2; SPS = sum score of all three HSPS-G subfactors; AES, aesthetic sensitivity; EOE, ease of excitation; LST, low sensory threshold; N, neuroticism; E, extraversion; O, openness; A, agreeableness; C, conscientiousness.

### Latent profile analysis

#### Class enumeration and class diagnostics

Table 4 presents the model fit indices for the models in Sample A, Sample B, and the total sample. When inspecting the indicator profiles (i.e., subfactor profiles) of the several *k* + 1 class model solutions, we noticed that, as the number of classes increased, distinct classes arose on the basis of fine differentiations in the AES probabilities. This finding can be informative and theoretically meaningful only to a certain extent. Therefore, we concluded that overfitting occurred from the 6-class solution onwards. Focusing on the pattern obtained for AES (i.e., the positive trait space) and EOE/LST (i.e., the negative trait space) seemed to offer an interesting qualitative differentiation beginning with the 4-class model solutions. Hence, we selected models with 4 to 6 classes as “candidate models” (Masyn, 2013). The relative fit indices (i.e., BLRT and VLMR LRT) were inconclusive, as all model comparisons showed a significant improvement (i.e., *k* compared with *k* + 1 classes). LST and EOE regularly revealed the highest BVRs due to their high zero-order correlation (*r* = 0.63, *p* < 0.01) (see Table 3). Therefore, direct effects were probed (Oberski, 2016) when the BVRs were larger than 4 (Magidson et al., 2020). However, the fit indices did not improve substantially.

**Table 4:** Model fit indices of Sample A, Sample B, and total sample.

#	LL	BIC	AIC	BVR	VLMR	*p*	CE	*R* ^2^	AWE	ICL-BIC
Sample A (*N* = 1,102)										
2	−9747.02	19585.11	19520.05	76.28	901.14	<0.001	0.07	0.71	20095.90	19965.84
3	−9633.85	19407.79	19307.69	21.75	226.35	<0.001	0.12	0.70	20215.82	20015.72
**4**	**−9451.49**	**19092.11**	**18956.98**	**18.26**	**364.71**	**<0.001**	**0.13**	**0.73**	**20086.97**	**19816.84**
5	−9387.09	19012.35	18842.18	14.43	128.80	<0.001	0.17	0.73	20232.23	19892.06
6	−9192.85	18672.90	18467.70	18.02	388.48	<0.001	0.15	0.79	19880.42	19470.22
Sample B (*N* = 526)										
2	−4552.19	9185.83	9130.38	19.86	244.94	<0.001	0.11	0.61	9583.95	9463.50
3	−4510.70	9146.70	9061.40	3.71	82.98	<0.001	0.12	0.65	9633.17	9447.86
**4**	**−4460.22**	**9089.60**	**8974.43**	**3.84**	**100.96**	**<0.001**	**0.12**	**0.72**	**9660.04**	**9409.88**
5	−4431.79	9076.61	8931.59	1.89	56.85	<0.001	0.16	0.70	9804.57	9489.55
6	−4362.76	8982.39	8807.51	5.84	138.07	<0.001	0.15	0.76	9757.67	9377.79
Total Sample (*N* = 1,628)										
2	−14344.88	28785.89	28715.76	101.33	1117.60	<0.001	0.09	0.66	29617.48	29482.34
3	−14059.89	28267.68	28159.78	74.67	569.98	<0.001	0.09	0.76	29188.79	28980.88
**4**	**−13883.63**	**27966.93**	**27821.26**	**28.85**	**352.51**	**<0.001**	**0.11**	**0.75**	**29178.93**	**28898.26**
5	−13552.85	27357.14	27173.71	32.11	661.56	<0.001	0.13	0.79	28721.20	28367.76
6	−13136.74	26576.67	26355.47	39.06	832.23	<0.001	0.10	0.85	27816.52	27390.32

#, number of classes; LL, LogLikelihood; BIC, Bayes Information Criterion; AIC, Akaike Information Criterion; BVR, maximum bivariate residuals; VLMR, Vuong-Lo–Mendell–Rubin Likelihood Ratio test value; p, type I error probability of VLMR; CE, classification error; *R*^2^, entropy; AWE, approximate weight of evidence; ICL-BIC, integrated completed likelihood. Boldly typed model fit indices highlight the final model.

**Figure 1:**
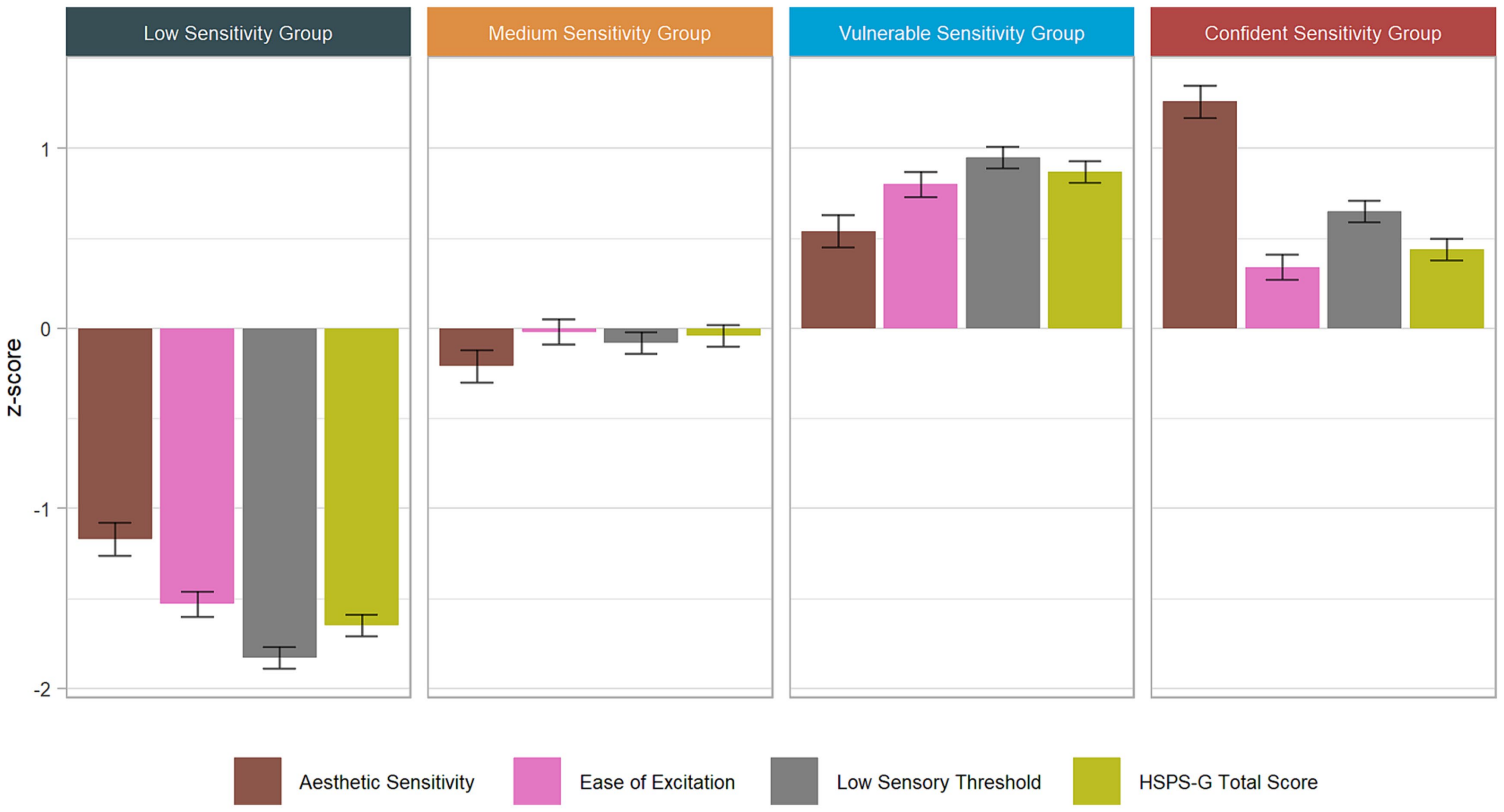
Standardized mean profiles of HSPS-G scores across sensitivity groups (total sample).

Below, we outline the process of assessing the candidate models for each sample separately and provide the model fit indices in Table 4. Figure 1 offers the HSPS profiling in the total sample. For Sample A, the 4-class and 5-class models exhibited similar Entropy (*R*^2^). The ICs (i.e., BIC and AIC) decreased only slightly when one more class was added; however, the CE appeared unfavorable compared with the 4-class model (13 to 17%). The 5-class and 6-class models showed decreases in the smallest class size to below 10 percent. The AWE and ICL-BIC increased from a 4-class to a 5-class solution, indicating that the best trade-off was achieved with the four classes. To resolve the remaining BVRs, we probed the impact of a direct effect on the relationship between LST and EOE. We could instead see a deterioration in the model fit indices (especially Entropy and CE) and yielded only slight superiority concerning the LogLikelihood (LL) and BIC. Therefore, we used the “native” (i.e., unconditional) model (Wang and Wang, 2020).

For sample B, the 4-class model showed a clear superiority over the 3-class and 5-class models with respect to the trade-off between Entropy and CE. The BVRs were within the recommended range. The 6-class model showed differentiation in the lower segment of the HSPS-G total score, which appeared uninformative, and class size comprised only 5 %. Again, the AWE and ICL-BIC increased from the 4-class to the 5-class solution. In Table 5, we present the AvePPs for each of the four classes of the total sample model. All groups reached the 0.70 threshold, indicating good separation.

**Table 5:** Average posterior probabilities (AvePP) of women, men and the total sample.

	LSG	MSG	VSG	CSG
**AvePP of the total sample**				
LSG	**0.90**	0.10	0.00	0.00
MSG	0.05	**0.89**	0.06	0.00
VSG	0.00	0.17	**0.83**	0.00
CSG	0.01	0.03	0.03	**0.93**
**AvePP of women**				
LSG	**0.89**	0.12	0.00	0.00
MSG	0.04	**0.89**	0.07	0.00
VSG	0.00	0.16	**0.84**	0.00
CSG	0.01	0.03	0.03	**0.93**
**AvePP of men**				
LSG	**0.93**	0.07	0.00	0.00
MSG	0.07	**0.90**	0.03	0.00
VSG	0.00	0.20	**0.80**	0.00
CSG	0.01	0.05	0.01	**0.94**

LSG, low sensitivity group; MSG, medium sensitivity group; VSG, vulnerable sensitivity group; CSG, confident sensitivity group; AvePP, average posterior probability. Diagonal values (bold) indicate the AvePP.

Ultimately, considering the profiles, model fit statistics, and substantive interpretations, the best coherence across the two samples was attained with a 4-class model solution. This solution unveiled a qualitative differentiation at the upper range of the HSPS-G total score distribution, marked by opposite patterns for the AES and EOE subfactors. Consequently, we opted for this 4-class model for subsequent personality characterization and interpretation.

### Mapping the sensitivity groups in the FFM space

**Figure 2:**
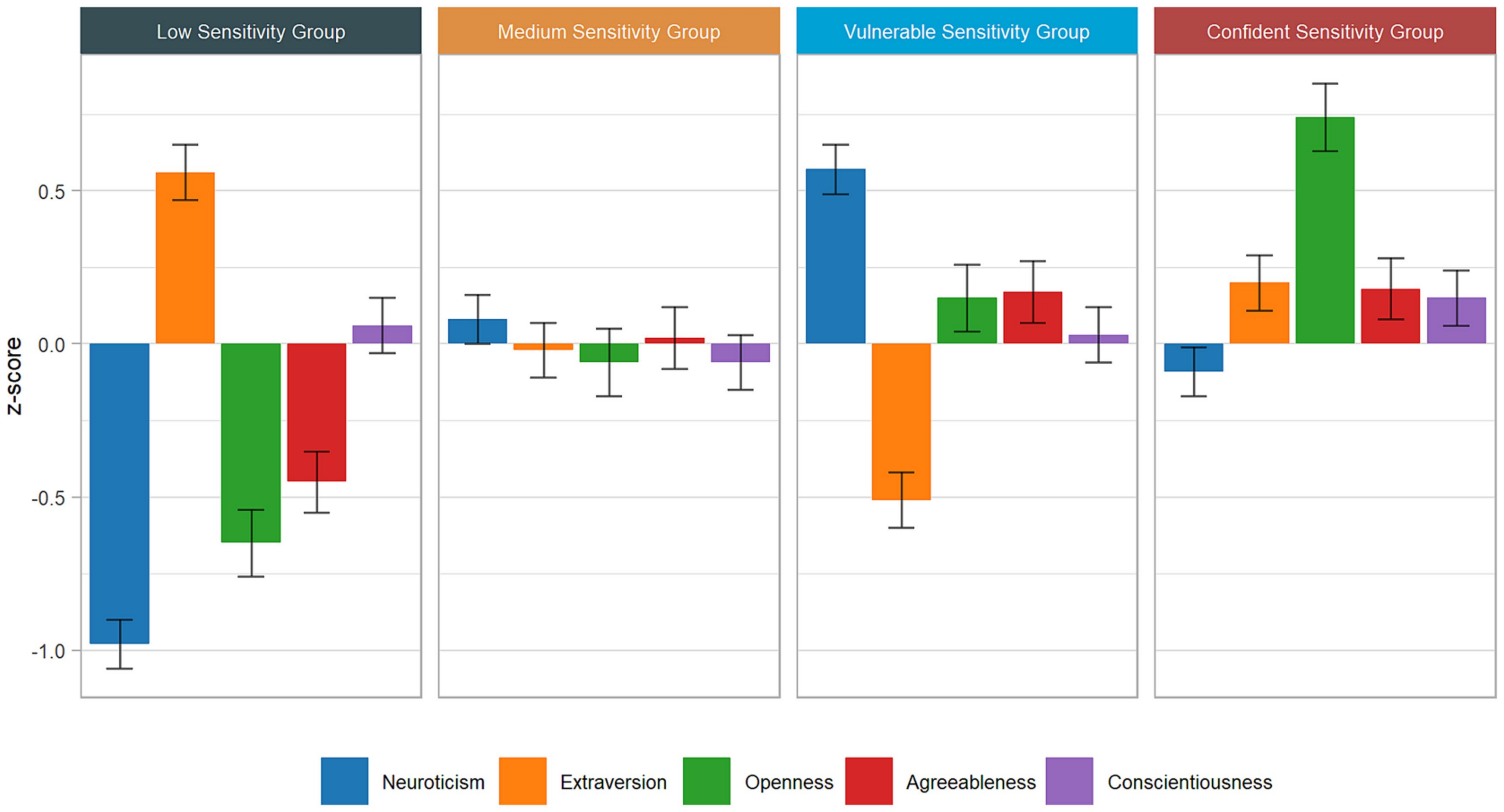
Standardized mean profiles of personality domains across sensitivity groups (Sample A).

**Figure 3:**
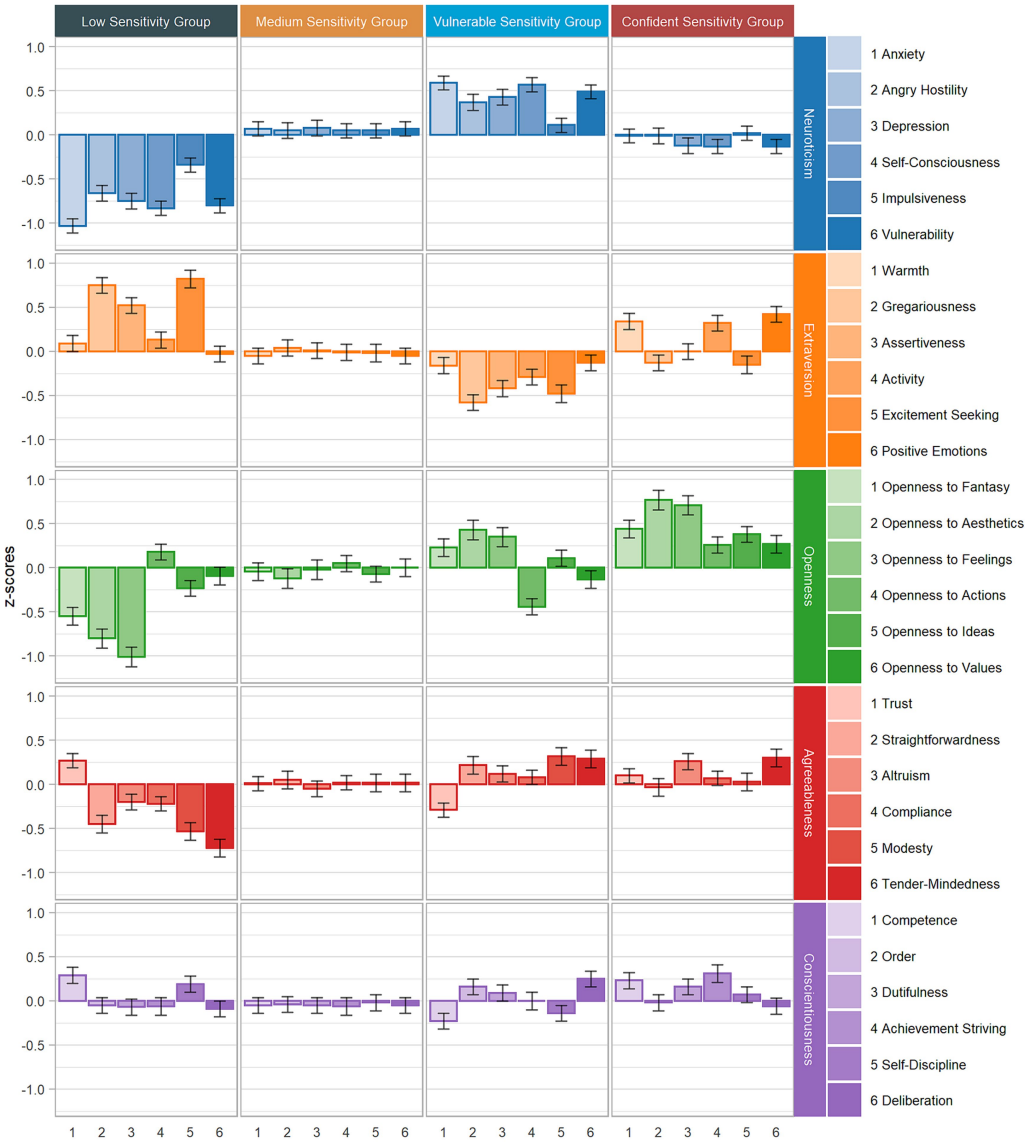
Standardized mean profiles of the personality facets across sensitivity groups (Sample A).

To succinctly present the extensive results, Figures 1–3 visually represent the characterizations of the sensitivity groups. These figures illustrate the standardized means of the HSPS-G subfactors as well as the FFM profiles, encompassing the FFM domains and facets across all sensitivity groups. The confidence intervals offer insights into which characteristics exhibit significant *post hoc* differences, along with their effect sizes. To enhance clarity with respect to subtle differences, Supplementary Table S1 complements the figures with specific values and indexes for *post hoc* distinctions. Deviations from average scores were considered when *z*-scores reached 0.2, and these are interpreted as being profile-defining. To do so, we relied on Cohen (1992) recommendations for small effect sizes Cohen (1992). In the following sections, we present the key findings that are essential for interpreting the profiles. First, we analyze the outcomes for the HSPS-G subfactors, FFM domains, and FFM facets across all sensitivity groups. Subsequently, we delve into qualitative comparisons between the two high sensitivity groups.

#### The profiles of the low sensitivity group

Profile 1 comprised 9–13% of the individuals and was characterized by markedly below-average scores on all three HSPS-G subfactors and the total score. We named this group the Low Sensitivity Group (LSG). At the domain level, this group exhibited below-average scores on Neuroticism, Openness, and Agreeableness, whereas its Extraversion score was above average. On the FFM facet level, this group displayed a relatively uniform pattern of below-average expressions on the Neuroticism facets, with Impulsiveness (N5) being the lowest. Conversely, the facet profile was more varied for the Extraversion facets, highlighting characteristics such as Gregariousness (E2), Assertiveness (E3), and Excitement-Seeking (E5). There was a consistent trend of below-average expression on the Openness facets: Fantasy (O1), Aesthetics (O2), Feelings (O3), and Ideas (O5). Trust (A1) exhibited above-average scores, whereas all the other facets in the Agreeableness domain were below average, with varying effect sizes. For the Conscientiousness facets, only Competence (C1) was slightly higher than average.

#### The profiles of the medium sensitivity group

Profile 2 included 40–60% of the individuals and was typified by moderate scores on all three HSPS-G subfactors, earning it the Medium Sensitivity Group (MSG) designation. As anticipated, this profile represented the HSPS means of the total sample. The FFM domain and facet scores all reflected average scores.

#### The profiles of the vulnerable sensitivity group

Profile 3 comprised 20–38% of individuals and was characterized by an above-average HSPS total score, primarily driven by the EOE and LST subfactors (predominantly in the negative trait space). The AES subfactor exhibited relatively lower scores in comparison with the fourth group, yet still above average. We labeled this third sensitivity group the Vulnerable Sensitivity Group (VSG). At the domain level, this group was marked by above-average Neuroticism scores and below-average Extraversion scores. All other domain mean scores were within the average range, although there was a variability in magnitude. Notably, the Openness and Agreeableness mean scores surpassed those of the Medium Sensitivity Group. At the facet level, the VSG demonstrated a relatively consistent pattern of above-average scores on the Neuroticism facets, with the exception of Impulsivity (N5). By contrast, the Extraversion facets revealed more diversity. Specifically, below-average scores on Gregariousness (E2), Assertiveness (E3), Activity (E4), and Excitement-Seeking (E5) were prominent. For the Openness facets, the group demonstrated above-average levels of Fantasy (O1), Aesthetics (O2), and Feelings (O3), whereas Actions (O4) fell below average. For Agreeableness, the Trust (A1) scores were below average, whereas the Straightforwardness (A2), Modesty (A5), and Tender-Mindedness (A6) scores were above average. For Conscientiousness, only Competence (C1) and Deliberation (C6) were distinct such that Competence exhibited below-average scores, whereas Deliberation was above average.

#### The profiles of the confident sensitivity group

Profile 4 comprised 10–12% of individuals and was characterized by an above-average HSPS total score, primarily influenced by the AES subfactor (predominantly in the positive trait space). The LST and EOE levels were notably lower in comparison with the Vulnerable Sensitivity Group. We named this fourth sensitivity group the Confident Sensitivity Group (CSG). At the domain level, this group displayed average scores on Neuroticism, Agreeableness, and Conscientiousness. Its Extraversion scores were above average, and the Openness domain stood out with mean scores that were significantly above average. On the facet level, the Neuroticism facet pattern closely resembled that of the Medium Sensitivity Group. For Extraversion, the facets of Warmth (E1), Activity (E4), and Positive Emotions (E6) all had above-average scores. All the Openness facets also registered as above average, but there were noteworthy differences among them. In particular, Fantasy (O1), Aesthetics (O2), and Feelings (O3) stood out, with O2 and O3 even showing somewhat large deviations from the mean. For Agreeableness, the facets of Altruism (A3) and Tender-Mindedness (A6) exhibited above-average scores. Finally, for Conscientiousness, the facets of Competence (C1) and Achievement-Striving (C4) demonstrated above-average scores.

### Contrasting the vulnerable sensitivity group and the confident sensitivity group

In Sample A, there were no significant differences between the CSG and the VSG in the HSPS total scores [Wald(1) = 0.71, *p* = 0.40] and the LST subfactor score [Wald(1) = 2.63, *p* = 0.11]. Thus, in Sample A, the distinction between these two sensitivity groups was derived solely from contrasting patterns in the AES and EOE subfactor scores. In the total sample, the effect size for the mean difference between the VSG and the CSG on the AES subfactor was moderate to large (*d* = 0.72). The effect sizes for the mean differences between the VSG and the CSG on the EOE and LST subfactors were small to medium (*d*_EOE_ = 0.46; *d*_LST_ = 0.43). The standardized mean subfactor scores from the total sample are presented in Figure 1, whereas all the raw means are presented in Table 6.

**Table 6:** Group sizes, HSPS-G raw means, and SE across all sensitivity groups and samples.

	LSG *M* (*SE*)	MSG *M* (*SE*)	VSG *M* (*SE*)	CSG *M* (*SE*)	Total *M* (*SE*)
**Sample A**					
Group sizes (%)	10	40	38	12	
AES	12.07(0.32)	15.84(0.11)	17.18(0.08)	20.00(0.00)	16.48(0.06)
EOE	18.87(0.57)	27.75(0.24)	33.56(0.17)	32.34(0.45)	29.61(0.14)
LST	14.29(0.59)	26.93(0.26)	35.05(0.21)^a^	34.18(0.49)^a^	29.60(0.16)
SPS	45.23(1.02)	70.52(0.37)	85.78(0.31)^a^	86.51(0.81)^a^	75.68(0.24)
**Sample B**					
group sizes (%)	9	60	21	10	
AES	14.46(0.55)	15.65(0.13)	18.19(0.10)	20.00(0.00)	16.51(0.10)
EOE	22.39(0.72)	30.54(0.24)	35.86(0.28)	31.91(0.61)	31.05(0.18)
LST	19.64(0.90)	29.68(0.31)^a^	37.01(0.33)	31.42(0.89)^a^	30.48(0.23)
SPS	56.50(1.36)	75.87(0.43)	91.06(0.47)	83.33(0.17)	78.04(0.33)
**Total sample**					
Group sizes (%)	13	55	20	12	
AES	13.23(0.25)	15.89(0.07)	18.01(0.06)	20.00(0.00)	16.49(0.05)
EOE	20.64(0.42)	29.94(0.15)	35.01(0.17)	32.19(0.36)	30.07(0.11)
LST	17.03(0.47)	29.55(0.18)	36.69(0.21)	33.33(0.43)	29.88(0.13)
SPS	50.91(0.80)	75.38(0.26)	89.71(0.29)	85.51(0.67)	76.44(0.20)

LSG, low sensitivity group; MSG, medium sensitivity group; VSG, vulnerable sensitivity group; CSG, confident sensitivity group. ‘a’ indicates no significant group mean differences; SPS, HSPS-G total score; AES, aesthetic sensitivity; LST, low sensory threshold; EOE, ease of excitation; AvePP, average posterior probability.

#### Neuroticism

The difference between the VSG and the CSG on Neuroticism corresponded to a medium effect size [Wald(1) = 30.29, *p* < 0.001; *d*_N_ = 0.66]. The VSG had the highest domain and facet scores. By contrast, the CSG’s Neuroticism pattern resembled that of the MSG. The CSG and the VSG contrasted across all facets except for Impulsiveness [N5; Wald(1) = 0.62, *p* = 0.43], which seemed to be the least distinctive facet. However, Vulnerability (N6) emerged as one of the most distinguishing facets across all the sensitivity groups. Notably, Anxiety (N1), Angry Hostility (N2), Depression (N3), Self-Consciousness (N4), and Vulnerability (N5) appeared to be particularly characteristic of the VSG. Whereas the effects were medium for Anxiety and Depression, the effects were smaller for Angry Hostility. Vulnerability and Self-Consciousness displayed the most pronounced effect sizes when the two high sensitivity groups were compared (*d*_N4_ = 0.70; *d*_N6_ = 0.62; all reported effects *p* < 0.01, except for N5).

#### Extraversion

For the Extraversion domain, the mean differences between the VSG and CSG was medium in magnitude [*d*_E_ = 0.71; Wald(1) = 38.40, *p* < 0.001]. The CSG showed some similarities with the LSG in exhibiting more Extraversion than the VSG. Specifically, the CSG had the highest scores on Warmth (E1), Activity (E4), and Positive Emotions (E6), whereas the VSG exhibited the lowest scores on these facets. The mean differences on the facets all fell within the medium range (*d*_E1_ = 0.50; *d*_E4_ = 0.61; *d*_E6_ = 0.55; all effects *p* < 0.001). Notably, the mean differences on Gregariousness (E2) and Assertiveness (E3) showed large effect sizes (*p* < 0.001). Both groups exhibited negative scores on Excitement-Seeking [N5; *d*_E5_ = 0.33; Wald(1) = 8.48, *p* < 0.01], yet the CSG’s pattern resembled that of the MSG.

#### Openness

For the Openness domain, the VSG was significantly different from the CSG, exhibiting a large effect size [*d*_O_ = 0.59; Wald(1) = 32.39, *p* < 0.001]. While the CSG recorded above-average scores, the VSG exhibited levels that were closer to the MSG. Only with respect to Fantasy (O1) did the CSG and VSG resemble each other [Wald(1) = 3.35, *p* = 0.07]. By contrast, for all other facets, the CSG surpassed the VSG (*d*_O2_ = 0.34; *d*_O3_ = 0.36; *d*_O4_ = 0.70; *d*_O5_ = 0.27, and *d*_O6_ = 0.4; all effects *p* < 0.001 except for O5 *p* = 0.03). Quantitatively, Actions (O4) appeared to be the most pertinent facet for distinguishing between the two high sensitivity groups.

#### Agreeableness

For the Agreeableness domain, there was no significant difference between the VSG and the CSG [Wald(1) = 0.01, *p* = 0.95]. However, small effects were observed on the facets of Trust (A1), Straightforwardness (A2), and Modesty (A5). On average, the CSG demonstrated higher levels of trustworthiness (*d*_A1_ = 0.39, *p* < 0.01), which resembled the MSG. Moreover, the CSG exhibited slightly lower scores on Straightforwardness (*d*_A2_ = 0.25, *p* = 0.04) compared with the VSG. Notably, the VSG displayed greater Modesty (*d*_A5_ = 0.29, *p* = 0.02) compared with the CSG, which had average levels similar to the MSG. No significant differences were found between the two sensitivity groups on Altruism (A3), Compliance (A4), or Tender-Mindedness (A6).

#### Conscientiousness

There were no significant differences between the VSG and the CSG on the Conscientiousness domain [Wald(1) = 0.86, *p* = 0.35]. By contrast, when considering the Conscientiousness facets, small effects were observed on the facets of Competence (C1), Achievement-Striving (C4), and Deliberation (C6). The CSG reported higher levels of self-efficacy and feelings of control over one’s life, with an effect size that was almost medium in magnitude (*d*_C1_ = 0.46, *p* < 0.001). Additionally, the CSG demonstrated higher levels of Achievement-Striving (*d*_C4_ = 0.31, *p* = 0.01). On the other hand, the VSG exhibited elevated levels of Deliberation (*d*_C6_ = 0.31, *p* = 0.02), whereas the CSG’s profile somewhat resembled that of the MSG.

### The influence of sex and age groups on the latent sensitivity groups

On the basis of the previous SPS literature (Ueno et al., 2019; Weyn et al., 2021, 2022; Trå et al., 2022; Liu et al., 2023; Pérez-Chacón et al., 2023; Sperati et al., 2024b), it seemed reasonable to assess the influence of sex and age groups on the 4-class sensitivity group model. The correlations found in the current study between the HSPS-G total score and age (*r* = 0.18, *p* < 0.01) and the HSPS-G total score and sex (*r* = 0.30, *p* < 0.01) were in line with previous these findings and justify controlling for any potential group effects. Controlling for potential group effects on the latent sensitivity group structure is best achieved with measurement invariance (MI) analyses. The MI analyses required for LPA models are conceptually comparable to those for confirmatory factor analysis (CFA; Masyn, 2017; Kankaraš et al., 2018).

Kankaraš et al. (2018) proposed a procedure for MI analyses for latent class models. The authors suggested a sequential practice that consists of estimating four consecutive models and applying a stepwise assessment of the relationship of the independent grouping variable (i.e., sex or age) with the latent model and the manifest model indicators. They proposed starting with the most heterogeneous model (i.e., Model A, unrestricted) and ending with the most homogenous model (i.e., Model D, most restricted model with no sex or age effects). Thus, we assessed all four models for the best model fit [the formal presentation of Models A to D in Kankaraš et al. (2018)].

First, we included sex as an independent grouping variable in the MI models (Models A to D). Table 7 presents the model indices. According to Kankaraš et al. (2018), the BIC is the most conclusive indicator of the best fitting model. In Model A, all model parameters were unrestricted (i.e., slope, intercept, and interaction terms) and were estimated for each sex separately as though each sex needed their own model. In Model B, the CRPs were allowed to be different; however, the slope parameters were assumed to be equal between the sexes. This model corresponds to metric equivalence in CFA (Kankaraš et al., 2018). Speaking in terms of an item analysis, the item difficulties of men and women can be different (see Supplementary Figures S1, S2). A previous study already demonstrated that men’s and women’s item difficulties are likely to be different when SPS is measured with the HSPS-G (Konrad and Herzberg, 2017). Moreover, the Highly Sensitive Child Scale frequently shows MI for sex groups (Weyn et al., 2021, 2022; Sperati et al., 2024b). In Model C, the CRPs (i.e., item difficulties) were restricted to be equal. In a CFA, loadings and item intercepts are assumed to be equal, corresponding to the notion of scalar equivalence. In Model D, we omitted the sex variable, corresponding to the model presented in Table 7 and assuming that sex had no influence on the latent modeling. Table 7 shows that Model B had the lowest BIC value. Therefore, metric equivalence could be accepted, thereby making it necessary to consider the different HSPS-G subfactor means for men and women separately. Consequently, in the following subchapters, we present the normative considerations and cut-off scores separately for men and women. Due to space constraints, we offer the sex-specific item-level difficulties in Supplementary Table S2 and Supplementary Figure S1. They show that (sex-specific) differential item functioning is most relevant for the items from the EOE subfactor.

**Table 7:** Model fit indices of the measurement invariance analyses regarding sex and age groups.

#	LL	BIC	AIC	Npar	VLMR	*p*	CE	*R* ^2^	AWE	ICL-BIC
**MI models regarding sex groups (*N* = 1,628)**										
A	−13766.30	27843.19	27616.60	42	–	–	0.12	0.74	29282.63	28846.04
**B**	**−13798.42**	**27840.87**	**27662.83**	**33**	**64.24**	**<0.001**	**0.12**	**0.74**	**29151.41**	**28808.37**
C	−13820.01	27861.87	27700.02	30	43.18	<0.001	0.11	0.75	29110.32	28798.46
D	−13883.63	27966.93	27821.26	27	127.25	<0.001	0.11	0.75	29178.93	28898.26
**MI models regarding age groups (*N* = 1,628)**										
A	−14044.53	28621.51	28233.07	72	–	–	0.18	0.65	30784.14	30035.69
B	−13841.12	28015.01	27772.23	45	406.83	<0.001	0.11	0.76	29394.25	28926.47
C	−14074.63	28415.48	28221.26	36	467.02	<0.001	0.18	0.65	30171.59	29797.36
**D**	**−13883.63**	**27966.93**	**27821.26**	**27**	**381.99**	**<0.001**	**0.11**	**0.76**	**29178.93**	**28898.26**

#, model denomination; LL, LogLikelihood; BIC, Bayes Information Criterion; AIC, Akaike Information Criterion; Npar, number of parameters; VLMR, Vuong-Lo–Mendell–Rubin Likelihood Ratio test value; p, type I error probability of VLMR; CE, classification error; *R*^2^, Entropy; AWE, approximate weight of evidence; ICL-BIC, integrated completed likelihood; we created four age categories (age groups range from 16 to 30; from 31 to 40; from 41 to 50; from 51 to 99). The fit indices of the final MI model are bold.

**Figure 4:**
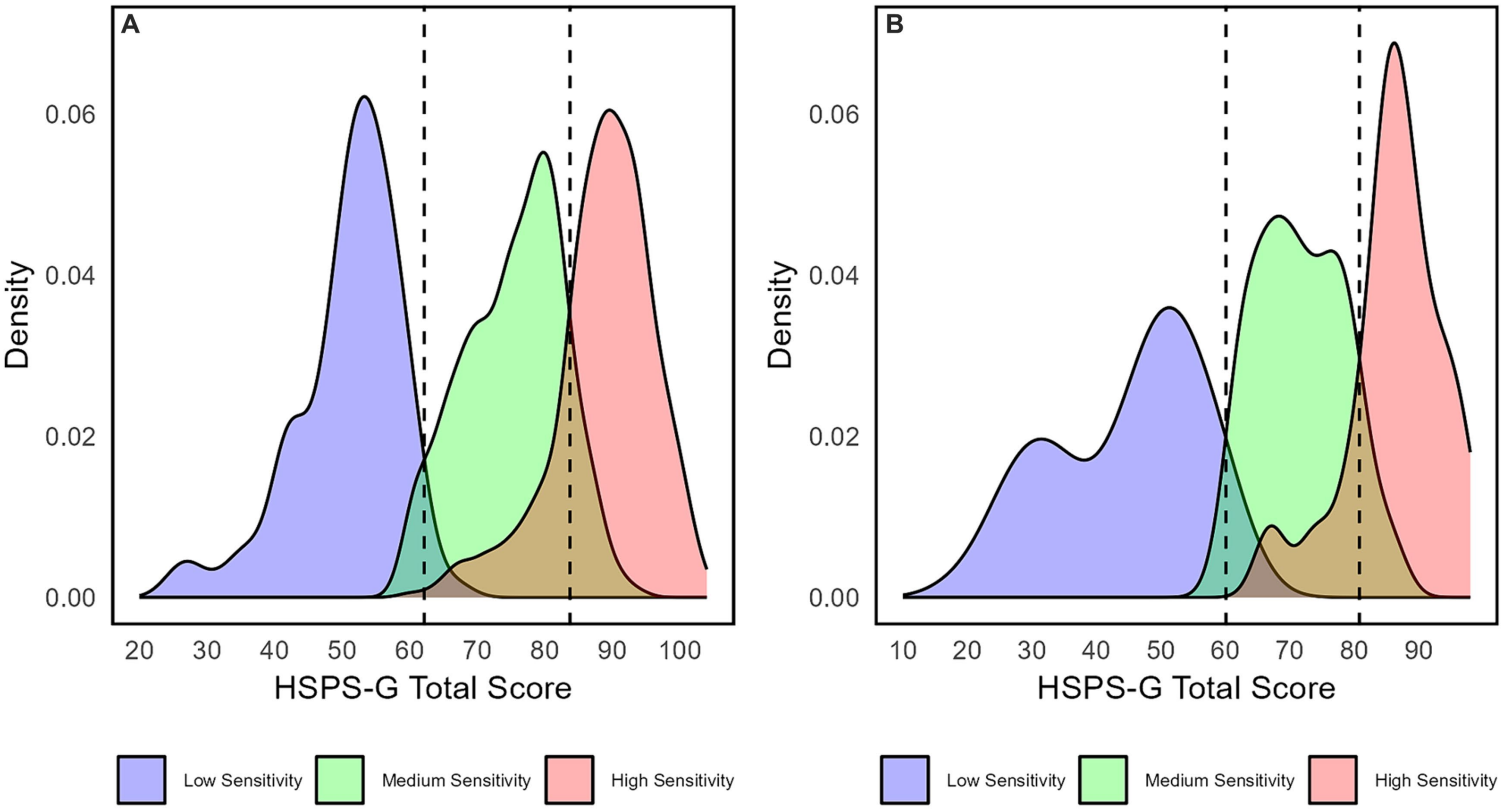
Sex-specific density plots and cut-off scores for **(A)** women and **(B)** men.

In line with the results of the MI analysis (i.e., partial homogeneity reflected by the best-fitting Model B), the sex-specific AvePP (Table 5) and density curves (Figure 4) supported the notion of structural equivalence. Table 5 shows that the classification of men and women (AvePP ranges from 0.80 to 0.94) from the 4-class model with four sensitivity groups was just as successful (Nagin, 2005). The most striking differences in the AvePP can be seen in the LSG and the VSG. Men were more accurately assigned to the LSG (AvePP = 0.93) than women (AvePP = 0.89). Conversely, the classification accuracy in the VSG was slightly higher for women (AvePP = 0.84) than for men (AvePP = 0.80).

Moreover, in Figure 4, we present the density curves of the three quantitative sensitivity ranges, thus enabling the derivation of the sex-specific cut-off scores. The overlapping proportions of the density curves represent the CE, whereas the overlap-free proportions reflect the AvePP. The highest classification inaccuracy appeared between the MSG and the two high-sensitivity groups (the VSG and the CSG). A closer look at the AvePP, depicted for all four sensitivity groups, allowed us to infer that issues in discriminating the VSG from the MSG were the main cause of the classification error (CE in the VSG in the total sample was 0.17 compared with 0.07 in the CSG).

Second, we included age groups as an independent variable in the MI models (Models A to D). The above description of consecutive restrictions on model parameters also applied to the second MI analysis with age. For this analysis, we created four age categories with nearly balanced sample sizes. In these groups, the ages ranged from 16 to 30 (*N* = 407), from 31 to 40 (*N* = 453), from 41 to 50 (*N* = 464), and from 51 to 99 (*N* = 304). In this analysis of the models with age, the best fitting model was Model D (i.e., complete homogeneity; see Table 7)—where the age groups did not influence the latent structure or the manifest indicators (Kankaraš et al., 2018).

In conclusion, our MI analyses revealed that it is vital to consider the sex-specific HSPS-G subfactor means when assigning men and women to the sensitivity groups because partial measurement invariance can be assumed for the latent profile model of sensitivity groups (due to the best fit of Model B). By contrast, it is most appropriate to assume that age does not influence the latent modeling (due to the best fit of Model D). Despite these findings from our exploratory study, the profiles for the CSG and the VSG based on the AES and EOE/LST subfactor constellation still held across all eight MI models, strengthening the validity of the distinction of latent sensitivity groups in the high-sensitivity range.

Finally, we showed that metric equivalence between men and women can be assumed for the sensitivity groups. Unfortunately, we could not transfer this finding to the external variables we included in our study (i.e., NEO-PI-R domains and facets). However, differences in levels of personality traits between the sexes have been identified (Bröhl et al., 2021). As our sample size lacks the power to test for potential mean effects, especially in the high-sensitivity groups, the question about whether there are sex-specific mean differences in the FFM personality domains and facets across the sensitivity groups is work for future research and thus needs to be considered a limitation of our findings.

### Cut-off scores and normative considerations

In a final secondary analysis, we aimed to provide other researchers and practitioners with the tools to utilize the sensitivity groups derived from our study in their own research or treatment settings. The sensitivity groups were technically derived from a discriminant function (DF) of the LPA, allowing the application to new datasets (Herzberg and Hoyer, 2009) or individual HSPS-G subfactor scores in two distinct ways. First, researchers can apply the DF directly to their data sets when their data includes the HSPS-G subfactors, which will be primarily relevant for researchers in German-speaking countries. Second, researchers and practitioners could use cut-off scores and *T* scores (i.e., normative values) to assign the observed individuals to the sensitivity groups. Here, we elucidate the latter (indirect) approach in more detail.

We graphically determined the threshold values of the total sample to derive cut-off scores, which mark the quantitative differences between the sensitivity groups (i.e., low, medium, high). Here, we combined the two high-sensitivity groups (i.e., the VSG and CSG) into one characteristic high-sensitivity range (see Figures 4A,B). We obtained scores of 60.10 (men) and 62.15 (women) for the separation of the LSG and MSG (i.e., lower cut-offs). Furthermore, scores of 80.80 (men) and 83.73 (women) emerged for the separation of the MSG and the high sensitivity range (i.e., upper cut-offs).

Now that we have achieved a quantitative distinction, we referred to the sex-specific normative values (Herzberg et al., 2022) for the qualitative differentiation in the high-sensitivity range, providing a normative approach for identifying the VSG and CSG (see Table 8). Given that sex differences have consistently been reported in the previous literature (Konrad and Herzberg, 2017; Weyn et al., 2021; Pérez-Chacón et al., 2023), a sex-specific approach was necessary. The categorization of test scores as below or above average by utilizing T scores of 40 (–1 SD) to 60 (+1 SD) as normative thresholds is a widely accepted practice (Cassel, 1963; Flanagan and Caltabiano, 2004). Table 8 presents the *T* scores applied to the total sample.

**Table 8:** Sex and sensitivity group-specific descriptives, group size, and *T* scores.

*N* (Men/Women)		Men			Women		
		*M*	*SD*	T score	*M*	*SD*	T score
LSG (74, 102)	HSP	44.69	11.55	42	50.87	7.84	36
	AES	12.05	3.40	42	13.40	3.69	39
	EOE	18.96	6.54	43	20.07	4.90	37
	LST	13.68	6.57	42	17.40	5.32	37
MSG (118, 806)	HSP	71.64	6.81	53	75.39	7.37	48
	AES	15.56	2.19	52	15.87	2.05	48
	EOE	29.53	4.42	53	29.84	4.53	49
	LST	26.55	4.78	52	29.68	5.31	49
VSG (24, 290)	HSP	89.42	4.56	63	90.74	4.27	61
	AES	18.21	0.83	59	18.08	0.90	54
	EOE	35.04	3.04	61	35.34	2.86	57
	LST	36.17	3.50	63	37.32	3.29	59
CSG (14, 200)	HSP	80.50	7.78	58	85.75	9.68	56
	AES	20.00	0.00	69	20.00	0.00	65
	EOE	29.36	4.13	52	32.36	5.19	52
	LST	31.14	6.65	57	33.40	6.16	53

LSG, low sensitivity group; MSG, medium sensitivity group; VSG, vulnerable sensitivity group; CSG, confident sensitivity group.

In the most unambiguous case of normative identification, the EOE/LST and AES subfactor scores would directly correspond with the normative categories (i.e., average, above-average, and below-average). Precisely speaking, a constellation of above-average scores on the AES subfactor accompanied by average scores on the EOE and LST subfactors would indicate the assignment of an individual to the CSG. And vice versa for the VSG. Unfortunately, this was only partly successful.

Suppose an individual surpasses the HSPS-G’s upper cut-off score (see above). In that case, the assignment to the VSG or CSG succeeds in three of four cases on the basis of the proposed categorization (i.e., relying on T40 and T60, as described above). For the female VSG group, however, the bounds deviate. The EOE and LST subfactors’ T scores were below 60. From our perspective, researchers could adapt their categorization to this exception by lowering the thresholds.

The disproportionately low number of items of the AES subfactor might have contributed to the lower HSPS total scores accompanied by the lower *T* scores in the CSG (*T*_male_ = 58 and *T*_female_ = 56 in the CSG compared with *T*_male_ = 63 and *T*_female_ = 61 in the VSG), considering that the AES was the dominant subfactor for the CSG. The probability of achieving above-average HSPS total score levels could increase with more positive items. Moreover, the T scores of the HSPS subfactors in the CSG reflected a more precise discriminant pattern than in the VSG, with the EOE and LST scores falling within the average ranges while the AES surpassed the average. Consequently, it will be more straightforward to use normative references to identify individuals in the CSG than in the VSG. This finding aligned with the other classification accuracy indices we reported above.

## Discussion

We applied an LPA to the HSPS subfactor level to identify latent sensitivity groups. In two independent samples, the four-class solution most consistently demonstrated the best fit to both data sets and enabled a meaningful interpretation. These four groups presented both a general quantitative distinction (i.e., low, medium, and high sensitivity) and qualitative differences within the high sensitivity spectrum (i.e., vulnerable high sensitivity and resilient/confident high sensitivity), the latter confirming our expectation of heterogeneity in the highly sensitive population. Next, to illuminate the differential personality functioning that is probably linked to the experience of sensitivity, we contrast vulnerable sensitivity (i.e., introverted-neurotic personality) and confident sensitivity (i.e., extraverted-open personality) in the light of previous research. First, we explain the labeling of our high sensitivity groups in line with prototypical personality research, as we use these labels throughout the article. Second, Extraversion appears to moderate the contrasting domain patterns of Neuroticism and Openness in the highly sensitive groups. These respective domain constellations appear to be relevant for individuals’ propensity to experience sensitivity as a blessing or as a challenge. In particular, we shed light on the social implications that come from viewing sensitivity through the lens of the FFM personality facets. Finally, we briefly discuss developmental ideas and the need for future studies with regard to the sensitivity groups.

The main reason we labeled the high sensitivity groups “vulnerable” and “confident” is that their profiles resembled well-established prototypical personality profiles and their associations, for example, with affective and cognitive wellbeing (Kerber et al., 2021). The VSG’s profile resembled that of a typical overcontroller (with slight deviations in the Openness domain), whereas the CSG’s profile was largely in line with the confident prototype (see Figure 2). The correspondence of the FFM domain profiles enabled us to integrate the differential empirical insights from prototypical personality research into our interpretation of the sensitivity groups’ profile. For instance, Kerber et al.’s (2021) study provided hints about the differential levels of locus of control, self-esteem, affective well-being, and cognitive well-being from a large representative German sample.

Regarding the VSG’s domain profile, the overcontrolled prototype tended to score above average on Neuroticism and below average on Extraversion. They often displayed traits such as constraint, emotional inhibition, external locus of control, low self-esteem, and diminished emotional and cognitive well-being, whereby all these features are linked to Neuroticism (Kerber et al., 2021). The VSG’s alignment with the overcontrolled prototype, coupled with their particularly elevated levels of Anxiety (N1), Self-Consciousness (N4), and Vulnerability (N6), indicates a neurotic personality structure that likely predisposes these individuals to psychopathological trajectories (Ormel et al., 2004; Griffith et al., 2010; Hengartner et al., 2016). In studies on SPS, this vulnerable predisposition has already been demonstrated, for instance, with respect to stress and burnout (Redfearn et al., 2020; Golonka and Gulla, 2021; Pérez-Chacón et al., 2021; Chacón et al., 2023), anxiety (Liss et al., 2005; Hofmann and Bitran, 2007; Liss et al., 2008), and depression (Liss et al., 2005; Wu et al., 2021). Conversely, the CSG’s domain profile is in line with the confident prototype, also resembling the well-adjusted prototype, typically scoring below-average on Neuroticism and showing above-average or intermediate levels on the other domains (Kerber et al., 2021), especially Extraversion and Openness (Herzberg and Hoyer, 2009). As children, confidents are easy and responsive in social interactions, love to do exercises, and can easily handle being separated from their parents (Kerber et al., 2021). In the Dunedin study, Caspi et al. (2003) found that the confident children appeared to score lowest on Traditionalism, highest in Social Potency, and highest on Positive Emotionality. These personality aspects were continuously expressed through high Extraversion and Openness scores at the age of 26. With respect to, for example, Warmth (E1), Activity (E4), Positive Emotions (E6), and Openness to Values (O6), the CSG’s personality facets matched the description of the confident prototype. Finally, confidents demonstrated at least average levels of internal locus of control, self-esteem, and positive emotional and cognitive well-being (Kerber et al., 2021), thereby contrasting the personality profile of the VSG.

Supplementing the impression of the prototypical personality with the respective differential associations in the nomological net, as demonstrated for the sensitivity groups’ dominant HSPS subfactors, creates a solid basis for labeling. More precisely, we found a highly sensitive group characterized by the EOE subfactor, which is anchored in the negative trait space, and a highly sensitive group characterized by the AES subfactor, embedded in the positive trait space. Hence, the differential associations of the dominant HSPS subfactors (i.e., EOE and AES) in the different sensitivity groups with external constructs in both the negative trait spaces [e.g., Negative Affect (Evans and Rothbart, 2009), alexithymia (Jakobson and Rigby, 2021)] and in the positive trait spaces (e.g., resilience and well-being; Sobocko and Zelenski, 2015; Gulla and Golonka, 2021) amplify the impression of vulnerability and confidence. Remarkably, Jauk et al. (2023) also found that the pattern of high EOE levels and lower AES levels (resembling the VSG) was associated with vulnerable narcissism and hypersensitive narcissism, which is linked to neurotic-introverted personality functioning. They used the original HSPS (Aron and Aron, 1997) and identified this constellation of AES and EOE as the group-determining subfactor pattern that underlines the reproducibility of our findings. In the following, we further emphasize and discuss the contrasting appearance of vulnerability and confidence with respect to the nuanced FFM facet profiles, among other characteristics.

No group had balanced levels of AES and EOE. Instead, it seems that adult HSPs, who have already had many learning experiences in life, with and within the environment, tend to develop an imbalanced typical interactive style (i.e., personality) and are inclined to face environmental stimuli with either above-average Neuroticism and less Openness (in the VSG) or with average Neuroticism and more Openness (in the CSG). This typological distinction represents the core contribution of our study because previous research has regularly found that SPS is strongly correlated with these domains (Lionetti et al., 2019; Bröhl et al., 2020; Pluess et al., 2023), thus implying validity for all participants in the sample from a variable-centered perspective. In addition, we observed that the level of Extraversion appears to be the sensitivity groups’ discriminant feature, which means that the HSPS subfactor pattern is mirrored by the individual level of Extraversion, thus determining which group an individual likely belongs to. Specifically, the CSG’s higher Extraversion levels are related to higher Openness and rather average levels of Neuroticism, whereas lower Extraversion (i.e., Introversion) is linked to higher levels of Neuroticism and rather average levels of Openness.

Examining the group-related patterns of Neuroticism and Openness allowed us to reflect on their respective potential for enlivening the blessings of SPS, as previous research has shown that the pattern of these domains predicts giftedness (Rinn et al., 2018; De Gucht et al., 2023) and abilities such as creativity (Bridges and Schendan, 2019) and interpersonal sensitivity (Tabak et al., 2022) in both ways (i.e., favoring or attenuating). Looking at the two domains separately, on the one hand, Neuroticism can dampen creativity and performance, similar to test anxiety in educational contexts, where individuals may struggle with limited working memory capacity due to anxiety, which ultimately impacts their performance (von der Embse et al., 2018; Hellwig and Roth, 2021). This phenomenon is supported by research on how working memory resources are strained by negative affect (similar to Neuroticism), which can undermine aesthetic experiences (Weigand and Jacobsen, 2021), thus potentially accounting for the slightly lower scores on Aesthetics (O2) in the VSG. On the other hand, the Openness domain predominantly emphasizes cognitive aspects and is often associated with intelligence (Gignac et al., 2004; Harris, 2004; Rammstedt et al., 2018) and creativity (Li et al., 2015; Puryear et al., 2017).

For instance, De Gucht et al. (2023) showed that gifted people score higher on the positive higher-order factor and lower on the negative higher-order factor of the Sensory Processing Sensitivity Questionnaire (SPSQ), which is a novel SPS measurement tool (De Gucht et al., 2022; De Gucht and Woestenburg, 2024). The effects of the mean differences were primarily driven by the Aesthetic Sensitivity and Social Affective Sensitivity subscales (both loading on the positive higher-order factor) and the subscale Emotional Physiological Reactivity (loading on the negative higher-order factor). Interestingly, in the construction analyses, when the authors demonstrated convergent validity between the traditional measure (i.e., HSPS) and the new one (i.e., SPSQ) (De Gucht et al., 2022), the most predictive SPSQ subscales (EPR and AS) of giftedness showed the highest significant correlations with the AES subfactor (correlating with AS, *r* = 0.66, *p* < 0.001) and the EOE subfactor (correlating with EPR, *r* = 0.83, *p* < 0.001). Furthermore, both SPSQ subscales showed the highest significant correlations with Neuroticism (for EPR, *r* = 0.70, *p* < 0.001) and Openness (for AS, *r* = 0.60, *p* < 0.001). Therefore, the authors probed the mediating roles of Neuroticism and Openness in the relationships between the SPSQ subscales with giftedness and found that the effects could partially be explained by Neuroticism and Openness, respectively. In conclusion, the study on giftedness supports the notion that the patterns of Neuroticism and Openness determine the potential for giftedness. Moreover, the close link between the latent factors of the two questionnaires suggests that the application of a person-centered approach using the SPSQ subscales could also reveal sensitivity groups that largely correspond to our VSG and CSG. However, in future studies, the use of six subscales, beyond the differentiation already provided by the three HSPS subfactors, could reveal more interindividual differences in the spectrum of high sensitivity.

Although the SPSQ’s Social Affective Sensitivity (SAS) subscale, which captures the social aspect of sensitivity (De Gucht et al., 2022), showed subordinate relevance for predicting giftedness (De Gucht et al., 2023), we think that this subscale could be a particularly interesting facet for a typological differentiation of high sensitivity in future research. In our study, the two sensitivity groups had equally high scores on Tender-Mindedness (i.e., reflecting an orientation toward the social and having sympathy for others, A6), indicating a shared trait with large effect sizes in mean differences when compared with the LSG (*d* = 1). Therefore, Tender-Mindedness seems to be a hallmark of high sensitivity when compared with low sensitivity. Nevertheless, the other parts of the facet profile suggest that the two sensitivity groups express social orientation in different ways (i.e., being compassionate, understanding, merciful, kind-hearted, people-friendly).

First, individuals in the VSG tend to be reserved, experience higher levels of worry (N1) and irritability, low self-confidence (N3), diminished self-worth (N3), reduced coping efficacy (N6), tend to strongly avoid big social gatherings or crowds (E2), are less assertive, do not like to engage in leadership behavior (E3), and prefer a slow-paced life (E4). This group’s profile also shows slightly lower Trust (A1) and more Modesty (A5), simultaneously showing a slightly below-average level of Competence (C1) and slightly more Deliberation (C6). When Deliberation is expressed more neurotically, which is evident in the VSG, it can easily turn into rumination and depression (Wisco, 1996). By contrast, individuals in the CSG seem to be the most cordial (E1), vigorous (E4), optimistic (E6), and most likely to express positive affect in comparison with all other sensitivity groups, even higher than the LSG in these regards. By extension, heightened levels of Actions (O4) indicate that the CSG is more engaged in trying new methods and willing to engage in new experiences and new surroundings. This group showed an average level of Trust (A1), slightly more Altruism (A3), higher Competence (C1), and more Achievement-Striving (C4). Considering both facet profiles allowed us to illuminate the fine-grained divergent colorations of Tender-Mindedness in the highly sensitive individuals, namely, neurotic-introverted in the VSG and extraverted-open in the CSG. Thus, the internalizing tendencies and passiveness in relation to SPS found by Bröhl et al. (2021), who took a variable-centered perspective, seem to apply to the VSG group but not the CSG. Person-centered approaches, such as the one we used in our study, allow such interindividual differences to be uncovered.

Second, both high sensitivity groups exhibited aspects of Openness, such as an active imagination, a penchant for daydreaming (O1), a strong interest in music and the arts (O2), and intense emotional experiences (O3). However, the VSG displayed lower levels of these traits, particularly in relation to recognizing and understanding emotions, as exemplified by the Feelings facet (O3). One explanation for this finding could be that individuals in the VSG tend to become overexcited and overwhelmed by sensory stimuli in earlier stages, consequently affecting their ability to fully appreciate the subtleties of emotional and social behavioral cues and resulting in a decline in their ability to emotionally connect with others and effectively regulate their own emotional experiences (Brindle et al., 2015; Sperati et al., 2024a). This idea is aligned with the notion of alexithymia and was recently investigated in relation to SPS (Jakobson and Rigby, 2021). In their study, the authors showed that the EOE subfactor was strongly related to issues in emotional appraisal (captured by the TAS-20 subscales Difficulties Identifying Feelings and Difficulties Differentiating Feelings), and thus, the VSG may also exhibit alexithymic tendencies. Moreover, in the second part of their study, Jakobson and Rigby (2021) showed that the two highly sensitive groups they found—alexithymic orchids (resembling the VSG) and lexithymic orchids (resembling the CSG)—differed in psychological health. The alexithymic orchids (i.e., high sensitivity linked to alexithymia) showed the highest psychological burdens in terms of depression, anxiety, and stress. This finding also illustrates a significant overlap with the overcontrolled prototype (Kerber et al., 2021), which we assume is also very similar to the VSG. Critically, Jakobson and Rigby (2021) used a different conceptualization of sensory processing in the LPA, which should not be confused with SPS, but has some overlap in measurement (Turjeman-Levi and Kluger, 2022). By contrast, the CSG demonstrated a more pronounced ability to recognize and understand emotions, which likely facilitates their utilization of the wealth of information provided by their keen emotional perceptiveness and depth of processing. For this group, the SPS element of overarousal seems to play a subordinate role, which is supported by previous results indicating that SPS is not inevitably linked to overarousal (Evans and Rothbart, 2008).

In conclusion, it is conceivable that the shared trait of Tender-Mindedness reflects the importance of the social environment for the highly sensitive person, but the remaining personality facets allude to a different interpersonal behavior and can supposedly be viewed in close connection to differential abilities in interpersonal sensitivity (Tabak et al., 2022). Tabak et al. (2022) showed that the EOE and LST subfactors and the AES subfactor had differential relationships with positive and negative interpersonal sensitivity (see Tabak et al., 2022, for more details).

In the early stages of life, the environment is predominantly a social experience for all people (e.g., Heim et al., 2019). However, highly sensitive people are disproportionately shaped by the environment from birth due to their genetic predisposition to sensitivity (Aron et al., 2012). Therefore, researchers have proposed that early experiences largely shape the type of environmental sensitivity an individual develops (vulnerable, neutral, vantage; see Pluess, 2015). The two different sensitivity groups that emerged in our analyses are closely related to this theory and to the seminal work by Aron and Aron (1997, Studies 2, 3, and 4), who also identified differences between sensitivity clusters in terms of (mal)adaptability, depending on the pattern of Neuroticism and Extraversion and probably due to different rearing environments. Later, the same authors showed that (early) negative childhood experiences are related to negative affectivity (which is strongly associated with neuroticism), especially when SPS is high (Aron et al., 2005). Moreover, as the EOE subfactor (dominant in VSG) seems to indicate a higher vulnerability potential (Jauk et al., 2023), and both alexithymia (Jakobson and Rigby, 2021) and narcissistic traits can be associated with or attributed to trauma (e.g., abuse) and a difficult upbringing, we assume that, in the VSG, the probability of a history of trauma with subsequent restrictions in emotion regulation is conclusive.

As our study did not include data on childhood characteristics (e.g., early parenting conditions), attachment style, or other biographical data, nor did we collect measures of reactivity, we can only guess about the origins of vulnerability and confidence. For the same reason, we can only conjecture about the type of environmental sensitivity (Pluess, 2015) in our sensitivity groups. However, at the beginning of our study, we assumed that a group with balanced scores on the HSPS subfactors representing the negative and positive sides of HSP would be most consistent with the theoretical concept of differential sensitivity. As a result of our exploratory approach, we were unable to find such a group, but differential susceptibility may apply to our groups.

At first glance, our naming of the sensitivity groups, particularly the label “vulnerable,” might imply agreement with the diathesis-stress model (Pluess, 2015). However, from our analyses, we cannot know whether the VSG would also benefit disproportionately from positive environments (i.e., vantage sensitivity), such as attending HSP support programs, seeking psychotherapy, or simply going to the forest and enjoying nature (Setti et al., 2022). Such an equally heightened reactivity to negatively and positively valenced environments would indicate differential susceptibility and would be consistent with the theory of SPS. Empirical findings are inconsistent in that some studies confirm the assumption of differential susceptibility (Slagt et al., 2015; Davies et al., 2021; Pluess et al., 2023), and some do not find empirical support (Slagt et al., 2017; Li et al., 2023). From a differential susceptibility perspective, it could be hypothesized that the CSG’s higher resilience is associated with vantage resistance, meaning that factors that determine resilience to adversity also lead to the CSG being less receptive to positive experiences (Pluess and Belsky, 2013). The question of ES type is the subject of current scientific debate, and future research should incorporate such variables to clarify the importance of rearing conditions for the development of a more vulnerable or more confident personality in the highly sensitive individual.

### Strengths and limitations

First, we employed two independent samples to cross-validate the latent sensitivity groups, a crucial step when utilizing LPA due to its data-driven nature. The identification of a latent group structure through LPA relies heavily on the sample’s composition (Herzberg and Roth, 2006). Consequently, the emergence of distinct subfactor patterns for highly sensitive groups, linked to either the negative or positive trait space of SPS, could have been influenced by self-selection biases in the samples (i.e., overrepresentation of self-identified highly sensitive people). Accordingly, the means in our sample were relatively high when compared with other studies conducted in the German language (Roth et al., 2023). In sample B and in the overall sample, we confirmed the prevailing prevalence values of approximately 30% of individuals with highly sensitivity, 40% with moderate sensitivity, and 30% with low sensitivity in the population (Lionetti et al., 2018; May et al., 2020; Yano and Oishi, 2021). Our findings also suggest that about 10% possess a confident (resilient) personality, whereas 20% could be considered vulnerable. It is essential to regard the sensitivity groups’ prevalence estimates, along with the associated cutoff-scores, as preliminary and in need of replication. To achieve greater reliability, it would be advisable to seek a representative sample or, at the very least, to ensure coverage of the general population. Such a sample was used for the construction and validation of the SPSQ (De Gucht et al., 2022). Collaborative research endeavors could yield a reliable population-valid discriminant function for assigning individuals to specific sensitivity groups. Such a discriminant function could prove valuable in smaller samples, which are common in laboratory studies or other research contexts in which the sample size necessary for person-centered exploratory methods such as LPA might not be attainable (Herzberg and Hoyer, 2009).

Second, the sex-specific correspondence of our latent group structure with German normative *T* scores demonstrated that the interpretation of the sensitivity groups holds substantial meaning. Consequently, we propose that, instead of focusing solely on the HSPS total score, an insightful approach would involve considering the subfactor patterns within the spectrum of high sensitivity. Unfortunately, our samples are characterized by a high proportion of women. Therefore, we conducted multigroup analyses to assess the effects of sex and age groups on latent group modeling. Age did not moderate the latent structure, however this result could be valid only for adults, as studies have observed effects in adolescent participants (Weyn et al., 2021). Due to the small number of male participants in the high-sensitivity groups (the VSG and CSG together yield *n* = 38), caution is warranted for the cut-off scores. Although we demonstrated their validity (i.e., metric equivalence), the validity is called into question by the imbalance between men and women (Yoon and Lai, 2018). In addition, there may be sex differences in the sensitivity groups for the FFM domain and facet profiles. Unfortunately, our sample size did not allow us to test for sex differences, as the (male) high-sensitivity groups were too small lacked power. Finally, future research could consider other sociodemographic variables, such as education or employment status, for multigroup analyses (i.e., measurement invariance analyses).

Lastly, the German AES subfactor contains only five items, has only moderate internal consistency (Cronbach’s *α* = 0.65), and the CSG shows no variance in this respect (*M* = 20; *SD* = 0). Therefore, a measurement tool that captures a broader picture of the HSP’s positive experiences could be beneficial. Recently, a novel extended questionnaire for adults that offers the opportunity to achieve differentiated insight into SPS’s positive and negative domains was proposed (De Gucht et al., 2022; De Gucht and Woestenburg, 2024). Our study yielded two sensitivity groups with different trait-space dominance, either EOE-dominant or AES-dominant. Using the SPSQ as a measurement tool could increase the probability of presenting a sensitivity group with balanced profiles.

## Conclusion

When combined with an average score on EOE, an above-average score on the AES subscale suggests classification as the confident type of highly sensitive person. These individuals utilize aesthetic impressions actively and extraverted with less neurotic coloration. From previous empirical literature on HSPS subfactors’ associations and prototypical personality types, we presume the confident type experiences heightened sensitivity more likely as a blessing. Furthermore, the confident inclination might be linked to greater self-worth and a superior capacity to self-regulate the intense processing of environmental stimuli compared to the vulnerable disposition. Conversely, an average score on the AES subfactor in conjunction with an above-average score on the EOE subfactor suggests classification as the vulnerable high-sensitivity type. These individuals adopt a passive and introverted approach to utilizing aesthetic impressions with more neurotic tendencies. From previous empirical literature on HSPS subfactors’ associations and prototypical personality types, we presume the vulnerable type experiences heightened sensitivity more likely as a challenge. Moreover, the vulnerable inclination might be tied to challenges in self-regulation of intense sensory processing, leading to heightened stress and health risks.

In summary, our study provides a more nuanced understanding of interindividual personality differences in highly sensitive individuals. Therefore, it is worthwhile to further investigate these differences to attain a more fine-grained and accurate knowledge of sensitivity effects, which might otherwise remain underexplored or subject to bias (Pluess, 2015; Acevedo, 2020).

## Data availability statement

The datasets presented in this study can be found in online repositories. The names of the repository/repositories and accession number(s) can be found below: https://osf.io/tycr9/?view_only=2a9a02c59f3c433592d95f4319dd82e8.

## Ethics statement

Ethical review and approval was not required for the study on human participants in accordance with the local legislation and institutional requirements. The studies were conducted in accordance with the local legislation and institutional requirements. The participants provided online informed consent to participate in this study.

## Author contributions

MB: Writing – original draft, Formal analysis, Data curation, Conceptualization. J-CM: Writing – review & editing, Visualization. PH: Methodology, Writing – review & editing, Supervision.
