# Supplementary material for: High sensitivity groups with distinct personality patterns: a person-centered perspective
Source: Front Psychol. 2024 Aug 16;15:1336474. doi: 10.3389/fpsyg.2024.1336474 (PMC11363424; doi:10.3389/fpsyg.2024.1336474)
Supplement: Supplementary file 1 [file Data_Sheet_1.PDF]

ESM TABLE 1. (Electronic Supplementary Material)

Overall effects and sensitivity group-specific post-hoc comparisons of personality domains and facets (including effect sizes).

	Sensitivity Groups						Effect sizes of group mean differences (Cohen's d)		
	LSG	MSG	VSG	CSG	overall				
	M(SE)	*M(SE)*	*M(SE)*	*M(SE)*	Wald (X^2)	*p*	VSG vs CSG	LSG vs VSG	LSG vs CSG
N	-0.98(.08)	0.08(.04)[a]	0.57(.07)	-0.09(.09)[a]	213.08	< .001	0.66	1.55	0.89
E	0.56(.09)	-0.02(.04)	-0.51(.08)	0.20(.08)	90.43	< .001	0.71	1.07	0.36
O	-0.65(.11)	-0.06(.04)	0.15(.07)	0.74(.07)	138.12	< .001	0.59	0.8	1.39
A	-0.45(.10)	0.02(.04)[a]	0.17(.08)[a]	0.18(.09)[a]	29.92	< .001	-	0.62	0.63
C	0.06(.09)[a,b]	-0.06(.04)[a]	0.03(.09)[a,b]	0.15(.09)[b]	5.12	0.16	-	-	-
N1	-1.03(.08)	0.07(.04)[a]	0.59(.07)	-0.01(.10)[a]	236.78	< .001	0.6	1.62	1.02
N2	-0.66(.09)	0.05(.04)[a]	0.37(.08)	-0.01(.10)[a]	76.80	< .001	0.38	1.03	0.65
N3	-0.75(.09)	0.08(.04)[a]	0.43(.07)	-0.12(.09)[a]	116.09	< .001	0.55	1.18	0.63
N4	-0.83(.08)	0.05(.04)[a]	0.57(.07)	-0.13(.09)[a]	178.12	< .001	0.7	1.4	0.7
N5	-0.34(.08)	0.05(.05)[a]	0.11(.08)[a]	0.02(.09)[a]	20.56	< .001	-	0.45	0.36
N6	-0.80(.08)	0.07(.04)	0.49(.08)	-0.13(.09)	143.52	< .001	0.62	1.29	0.67
E1	0.09(.09)[a]	-0.05(.04)[a,b]	-0.16(.09)[b]	0.34(.09)	19.81	< .001	0.5	0.25	0.25
E2	0.75(.09)	0.04(.04)[a]	-0.58(.07)	-0.13(.09)[a]	145.65	< .001	0.45	1.33	0.88
E3	0.52(.09)	0.01(.04)[a]	-0.42(.08)	-0.00(.09)[a]	62.76	< .001	0.42	0.94	0.52
E4	0.13(.09)[a,b]	-0.01(.04)[a]	-0.29(.08)	0.32(.10)[b]	24.46	< .001	0.61	0.42	-
E5	0.82(.10)	-0.02(.04)[a]	-0.48(.07)	-0.15(.08)[a]	117.79	< .001	0.33	1.3	0.97
E6	-0.03(.09)[a]	-0.05(.05)[a]	-0.13(.08)[a]	0.42(.08)	29.57	< .001	0.55	0.1	0.45
O1	-0.55(.10)	-0.04(.04)	0.23(.08)[a]	0.44(.08)[a]	73.25	< .001	-	0.78	0.99
O2	-0.80(.11)	-0.12(.04)	0.43(.07)	0.77(.06)	249.22	< .001	0.34	1.23	1.57

O3	-1.01(.11)	-0.02(.04)	0.35(.07)	0.71(.06)	239.91	< .001	0.36	1.36	1.72
O4	0.18(.09)^{a,b}	0.05(.04)^{b}	-0.44(.08)	0.26(.10)^{a}	39.39	< .001	0.7	0.62	-
O5	-0.23(.09)^{a}	-0.07(.04)^{a,b}	0.11(.09)^{b}	0.38(.08)	33.11	< .001	0.27	0.34	0.61
O6	-0.09(.10)^{a}	0.00(.04)^{a}	-0.13(.09)^{a}	0.27(.09)	12.73	.005	0.4	-	0.36
A1	0.27(.08)^{a}	0.01(.04)^{b}	-0.29(.08)	0.10(.10)^{a,b}	23.48	< .001	0.39	0.56	-
A2	-0.45(.10)	0.05(.04)^{a,b}	0.22(.08)^{a}	-0.03(.09)^{b}	31.91	< .001	0.25	0.67	0.42
A3	-0.20(.09)^{a}	-0.05(.04)^{a,b}	0.12(.08)^{b,c}	0.26(.09)^{c}	16.20	.001	-	0.32	0.46
A4	-0.22(.08)	0.02(.04)^{a}	0.08(.08)^{a}	0.07(.10)^{a}	8.40	.03	-	0.3	0.29
A5	-0.53(.10)	0.02(.04)^{a}	0.32(.08)	0.03(.09)^{a}	47.27	< .001	0.29	0.85	0.56
A6	-0.72(.10)	0.02(.04)	0.29(.08)^{a}	0.30(.08)^{a}	77.15	< .001	-	1.01	1.02
C1	0.29(.09)^{a}	-0.05(.04)^{b}	-0.23(.09)^{b}	0.23(.09)^{a}	24.80	< .001	0.46	0.52	-
C2	-0.05(.09)^{a}	-0.04(.04)^{a}	0.16(.09)^{a}	-0.02(.09)^{a}	3.58	.31	-	-	-
C3	-0.07(.09)^{a,b,c}	-0.05(.04)^{a,c}	0.09(.08)^{a,b,c}	0.16(.09)^{b,c}	6.13	.11	-	-	-
C4	-0.06(.10)^{a}	-0.06(.04)^{a}	-0.00(.09)^{a}	0.31(.09)	15.14	.002	0.31	-	0.37
C5	0.19(.09)^{b,c}	-0.02(.04)^{a,c}	-0.14(.09)^{a,c}	0.07(.09)^{c}	7.92	.048	-	0.33	-
C6	-0.09(.09)^{a}	-0.05(.04)^{a}	0.25(.09)	-0.06(.09)^{a}	10.34	.016	0.31	0.34	-

Note. Depicted values are standardized means; Shared indices indicate no group differences; *M* = Mean; *SE* = Standard error; LSG = Low Sensitivity Group; MSG = Medium Sensitivity Group; VSG = Vulnerable Sensitivity Group; CSG = Confident Sensitivity Group.
