# Supplementary material for: High sensitivity groups with distinct personality patterns: a person-centered perspective
Source: Front Psychol. 2024 Aug 16;15:1336474. doi: 10.3389/fpsyg.2024.1336474 (PMC11363424; doi:10.3389/fpsyg.2024.1336474)
Supplement: Supplementary file 2 [file Data_Sheet_2.PDF]

ESM Figure 1. Sex-specific response probabilities (proportions) of the 26 HSPS-G items across the sensitivity groups.

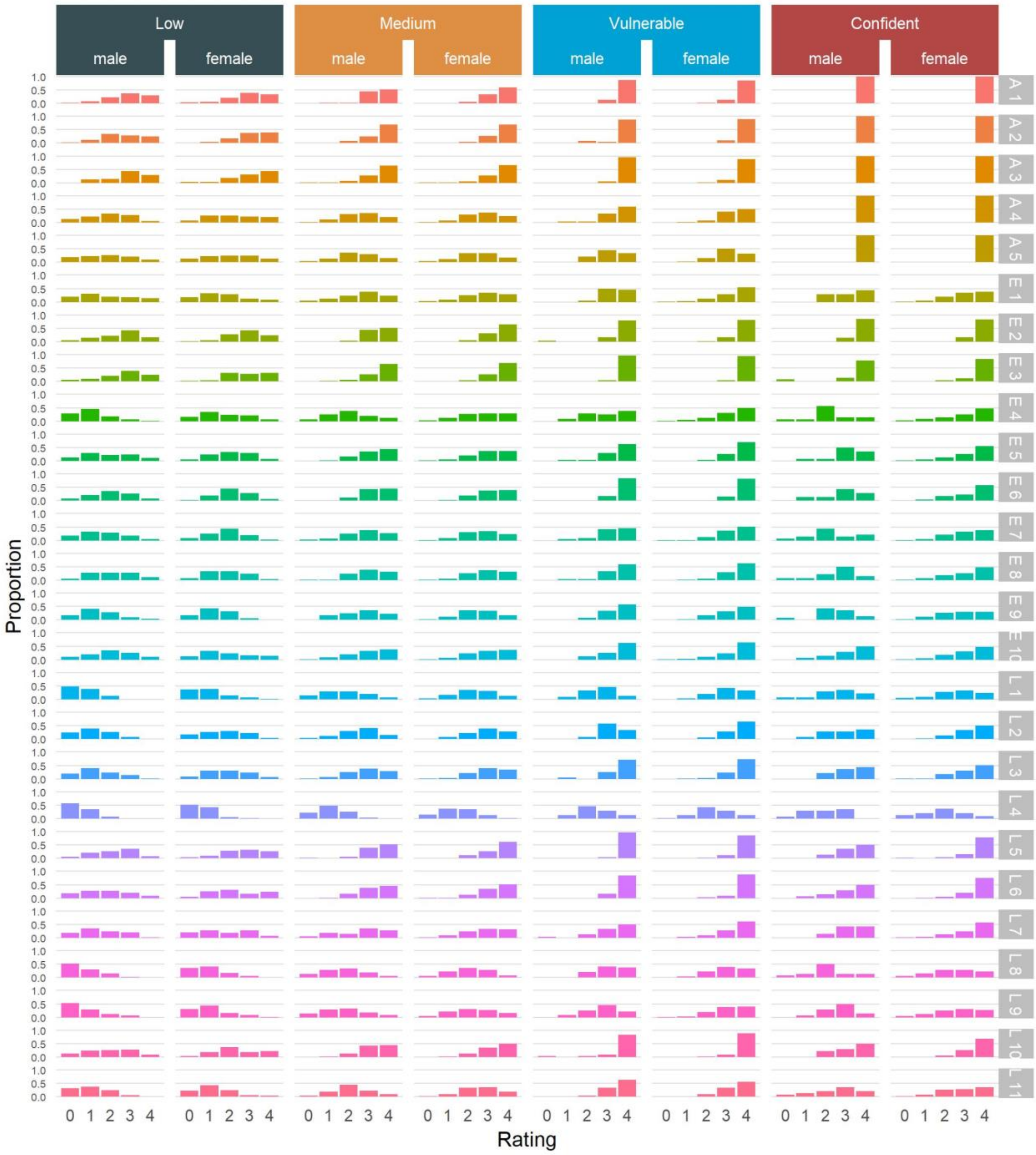

Note. A = items belong to the AES subfactor; E = items belong to the EOE subfactor; L = items belong to the LST subfactor; Items are sorted by subfactor and in order of appearance within the subfactor.
