# Supplementary material for: High sensitivity groups with distinct personality patterns: a person-centered perspective
Source: Front Psychol. 2024 Aug 16;15:1336474. doi: 10.3389/fpsyg.2024.1336474 (PMC11363424; doi:10.3389/fpsyg.2024.1336474)
Supplement: Supplementary file 3 [file Data_Sheet_3.PDF]

ESM Table 2. Sex-specific response probabilities of the 26 HSPS-G items across the sensitivity groups.

	Low Sensitivity Group				Medium Sensitivity Group				Vulnerable Sensitivity Group				Confident Sensitivity Group			
	Men (*N* = 74)		Women (*N* = 102)		Men (*N* = 118)		Women (*N* = 806)		Men (*N* = 24)		Women (*N* = 290)		Men (*N* = 14)		Women (*N* = 200)	
HSPS-G items	*M*	*SE*	*M*	*SE*	*M*	*SE*	*M*	*SE*	*M*	*SE*	*M*	*SE*	*M*	*SE*	*M*	*SE*
A 1	2.86	0.11	2.92	0.10	3.45	0.06	3.51	0.02	3.88	0.07	3.84	0.02	4.00	0.00	4.00	0.00
A 2	2.61	0.12	3.14	0.09	3.61	0.06	3.64	0.02	3.79	0.12	3.88	0.02	4.00	0.00	4.00	0.00
A 3	2.91	0.11	3.10	0.10	3.52	0.07	3.58	0.02	3.96	0.04	3.86	0.02	4.00	0.00	4.00	0.00
A 4	1.89	0.13	2.24	0.12	2.60	0.09	2.71	0.04	3.46	0.16	3.40	0.04	4.00	0.00	4.00	0.00
A 5	1.80	0.15	2.02	0.12	2.39	0.10	2.44	0.04	3.12	0.15	3.10	0.05	4.00	0.00	4.00	0.00
E 1	1.76	0.15	1.63	0.12	2.62	0.10	2.78	0.04	3.42	0.12	3.34	0.05	3.14	0.23	3.04	0.07
E 2	2.49	0.13	2.84	0.09	3.47	0.06	3.58	0.02	3.67	0.18	3.79	0.03	3.86	0.10	3.83	0.03
E 3	2.65	0.13	2.84	0.10	3.56	0.06	3.62	0.02	3.96	0.04	3.94	0.02	3.57	0.29	3.81	0.03
E 4	1.05	0.11	1.66	0.11	2.06	0.10	2.66	0.04	2.92	0.21	3.21	0.06	2.21	0.28	3.04	0.08
E 5	1.89	0.14	2.08	0.10	3.19	0.08	3.03	0.03	3.50	0.16	3.65	0.03	3.14	0.23	3.26	0.07
E 6	2.04	0.12	2.17	0.09	3.30	0.07	3.12	0.03	3.83	0.08	3.78	0.03	2.86	0.27	3.34	0.06
E 7	1.58	0.13	1.86	0.09	2.81	0.09	2.70	0.03	3.29	0.16	3.37	0.04	2.29	0.32	3.00	0.07
E 8	2.11	0.13	1.83	0.10	2.97	0.09	2.88	0.03	3.46	0.16	3.54	0.04	2.57	0.29	3.12	0.07
E 9	1.42	0.12	1.29	0.09	2.63	0.09	2.51	0.03	3.50	0.13	3.25	0.05	2.50	0.27	2.76	0.08
E 10	2.04	0.13	1.88	0.12	2.94	0.10	2.95	0.03	3.50	0.15	3.48	0.05	3.21	0.26	3.18	0.06

L 1	0.66	0.08	0.96	0.10	1.75	0.10	2.30	0.04	2.62	0.17	3.07	0.05	2.57	0.31	2.60	0.08
L 2	1.23	0.11	1.70	0.11	2.53	0.09	2.87	0.03	3.25	0.12	3.59	0.04	2.93	0.27	3.32	0.06
L 3	1.36	0.12	1.87	0.11	2.87	0.09	3.04	0.03	3.62	0.15	3.68	0.03	3.21	0.21	3.30	0.06
L 4	0.51	0.08	0.56	0.06	1.11	0.07	1.51	0.03	2.42	0.18	2.40	0.06	1.93	0.27	1.92	0.08
L 5	2.18	0.13	2.65	0.11	3.41	0.07	3.47	0.03	3.96	0.04	3.82	0.03	3.36	0.20	3.68	0.05
L 6	1.74	0.14	2.31	0.12	3.25	0.07	3.35	0.03	3.83	0.08	3.86	0.02	3.21	0.26	3.69	0.04
L 7	1.50	0.12	1.75	0.12	2.63	0.11	2.84	0.04	3.25	0.20	3.45	0.05	3.29	0.19	3.31	0.07
L 8	0.73	0.11	0.97	0.09	1.75	0.10	2.10	0.04	3.17	0.16	3.02	0.05	2.14	0.29	2.46	0.08
L 9	0.73	0.11	1.03	0.09	1.79	0.10	2.27	0.04	2.79	0.18	3.12	0.05	2.71	0.22	2.65	0.08
L 10	1.99	0.14	2.36	0.11	3.29	0.07	3.34	0.03	3.67	0.19	3.88	0.02	3.29	0.22	3.62	0.04
L 11	1.11	0.11	1.28	0.10	2.18	0.09	2.60	0.03	3.58	0.12	3.46	0.04	2.50	0.33	2.86	0.08

Note. A = items of the Aesthetic Sensitivity subfactor (AES); E = items of the Ease of Excitation subfactor (EOE); L = items of the Low Sensory Threshold subfactor (LST).
